# Multimodal intervention in 8- to 13-year-old French dyslexic readers: Study protocol for a randomized multicenter controlled crossover trial

**DOI:** 10.1186/s12887-022-03701-8

**Published:** 2022-12-28

**Authors:** Karine Louna Harrar-Eskinazi, Bruno De Cara, Gilles Leloup, Julie Nothelier, Hervé Caci, Johannes C. Ziegler, Sylvane Faure

**Affiliations:** 1 Laboratoire d’Anthropologie et de Psychologie Cliniques, Cognitives et Sociales (LAPCOS), Université Côte d’Azur, Campus Saint Jean d’Angély/MSHS Sud-Est, 3 Boulevard François Mitterrand, 06357 Nice, Cedex 4 France; 2 Centre Hospitalier Universitaire, Nice-CHU-Lenval, Nice, France; 3 Université Côte d’Azur, CoBtek, Nice, France; 4 Aix-Marseille Univ, CNRS, LPC, Marseille, France

**Keywords:** Dyslexia, Multimodal intervention, Underlying cognitive deficits, Randomized crossover clinical trial, Protocol

## Abstract

**Background:**

Developmental dyslexia, a specific and long-lasting learning disorder that prevents children from becoming efficient and fluent readers, has a severe impact on academic learning and behavior and may compromise professional and social development. Most remediation studies are based on the explicit or implicit assumption that dyslexia results from a single cause related to either impaired phonological or visual-attentional processing or impaired cross-modal integration. Yet, recent studies show that dyslexia is multifactorial and that many dyslexics have underlying deficits in several domains. The originality of the current study is to test a remediation approach that trains skills in all three domains using different training methods that are tailored to an individual’s cognitive profile as part of a longitudinal intervention study.

**Methods:**

This multicenter randomized crossover study will be conducted in three phases and will involve 120 dyslexic children between the ages of 8 and 13 years. The first phase serves as within-subject baseline period that lasts for 2 months. In this phase, all children undergo weekly speech-language therapy sessions without additional training at home (business-as-usual). During the second phase, all dyslexics receive three types of intensive interventions that last 2 month each: Phonological, visual-attentional, and cross-modal. The order of the first two interventions (phonological and visual-attentional) is swapped in two randomly assigned groups of 60 dyslexics each. This allows one to test the efficacy and additivity of each intervention (against baseline) and find out whether the order of delivery matters. During the third phase, the follow-up period, the intensive interventions are stopped, and all dyslexics will be tested after 2 months. Implementation fidelity will be assessed from the user data of the computerized intervention program and an “intention-to-treat” analysis will be performed on the children who quit the trial before the end.

**Discussion:**

The main objective of this study is to assess whether the three types of intensive intervention (phase 2) improve reading skills compared to baseline (i.e., non-intensive intervention, phase 1). The secondary objectives are to evaluate the effectiveness of each intervention and to test the effects of order of delivery on reading intervention outcomes. Reading comprehension, spelling performance and reading disorder impact of dyslexic readers are assessed immediately before and after the multimodal intervention and 2 months post-intervention.

**Trial registration:**

ClinicalTrials.gov, NCT04028310. Registered on July 18, 2019.

**Supplementary Information:**

The online version contains supplementary material available at 10.1186/s12887-022-03701-8.

## Administrative information

A SPIRIT-Checklist with the page numbers corresponding to each item is available in the Additional file 1.

**Table:** 

Title {1}	Multimodal intervention in 8-to 13-year-old French: Study protocol for a randomized multicenter controlled crossover trial.
Trial registration {2a and 2b}	ClinicalTrials.gov: NCT04028310. Registered on July 18, 2019.
Protocol version {3}	Version identifier is no. ID RCB 2019-A01453–54, 19-HPNCL-02, 07/18/2019
Author details {5a}	Karine, Louna Harrar-Eskinazi, PhD student LAPCOS laboratory, UCA-MSHS, France University Hospital Center, Nice-CHU-Lenval, France karine.harrar@etu.univ-cotedazur.fr Pr Sylvane FAURE LAPCOS laboratory, UCA-MSHS, France sylvane.faure@univ-cotedazur.fr Dr. Johannes C. Ziegler Director of the Cognitive Psychology Laboratory (UMR 7290) Director of the Institute of Language, Communication and the Brain (ILCB); CNRS and Aix-Marseille University, France johannes.ziegler@univ-amu.fr Dr. Bruno De Cara, Lecturer LAPCOS laboratory, UCA-MSHS, France bruno.de-cara@univ-cotedazur.fr Dr. Gilles Leloup, PhD University Hospital Center, Nice-CHU-Lenval, France COBTEK laboratory (7276), UCA-INRIA, France gileloup@gmail.com Dr. Hervé Caci University Hospital Center, Pediatric Hospitals of Nice-CHU-Lenval, France caci.h@pediatrie-chulenval-nice.fr Julie Nothelier, Research Assistant Cognitive Psychology Laboratory LPC; CNRS-Aix Marseille University, France julie.nothelier@gmail.com
Name and contact information for the trial sponsor {5b}	Trial sponsor**:** Nice-Lenval University Hospital Center Sponsor’s Reference: 19-HPNCL-02 Contact name**:** Dr. Hervé Caci, Dr. Dominique Donzeau, DRCI Address**:** 57 Avenue de la Californie, 06200 Nice Telephone**:** 00334 92 03 08 49; 00334 92 0 34,560 Email**:** caci.h@chu-nice.fr**;** donzeau.d@chu-nice.fr
Role of sponsor {5c}	The trial sponsor and funders have no influence on the planning, conduct and the design of this trial and will not have any role during its execution, analyses, interpretation of the data, or decision to submit results.

## Background {6a}

Developmental dyslexia is defined as a severe and persistent impairment that affects the capacity to learn to read despite normal intelligence and in the absence of neurological or psychiatric pathology, visual and auditory sensory deficits, or severe socio-educational deficiencies [1]. This neurodevelopmental disorder is characterized by various underlying neurocognitive deficits [2] and results in an underactivation of the reading network hosted in left occipito-temporo-parietal brain areas, most likely due to abnormalities in connectivity between these areas and more frontal and/or parietal language areas [3, 4]. Dyslexic readers encounter great difficulties in learning and automatizing the decoding of printed words [5], which consists in linking the basic units of written language (letters or graphemes) to the basic units of spoken language (sounds or phonemes). Reading fluency often remains difficult for children with dyslexia and requires a higher cognitive effort in comparison to normally developing children [6, 7]. The prevalence of developmental dyslexia is between 5 and 17% depending on cut-off criteria and language [6]. Dyslexia has severe repercussions on all aspects of school learning, self-esteem and professional development, which makes it a major public health issue [8]. Problems with reading, spelling and associated cognitive functions persist into adulthood [9].

For more than a century, many explanatory theories have been put forward for this complex learning disability without a true consensus ever being reached [10]. Currently, the scientific literature increasingly emphasizes the multifactorial nature of developmental dyslexia at the neurobiological [11, 12], cognitive [13–16], behavioral [17, 18] and environmental level [19]. Family studies indicate an uncertain etiology involving a complex interaction between biological, cognitive, behavioral and environmental factors [20]. That is, biological factors influence cognitive factors, which in turn influence behavior, each one being influenced by an individual’s environment [21]. Moreover, reading involves underlying multimodal cognitive processes that require the interplay of linguistic, visual, phonological, attentional and executive processes.

Developmental dyslexia is defined in current classifications, such as *The International Classification of Diseases (ICD-11) and The Diagnostic and Statistical Manual of Mental Disorders (DSM-5)*, mainly as a specific cognitive impairment of written language [1, 22]. These definitions, which focus on reading and spelling disorders, do not take into account underlying cognitive deficits (UCD) when making a diagnosis. Furthermore, the pathological behavioral manifestations in reading and the UCD may be different for each dyslexic reader. Thus, this complex multifactorial interaction leads not only to a nosographic and etiological multimodality but also to a symptomatologic and diagnostic multimodality. Developmental dyslexia is thus conceptualized as a “multifunctional deficit” [11, 23] or “multifaceted, heterogeneous disorder” [2], in accordance with a multifactorial causal hypothesis [24, 25], involving several UCD, themselves underpinned by the dysfunction of a large circuit of interconnected neural networks [26].

Three main theoretical hypotheses currently guide research on therapeutic interventions of developmental dyslexia: The phonological hypothesis, the visual-attentional hypothesis, and the cross-modal hypothesis. According to the phonological hypothesis, a specific alteration of phonological representations or poor access to phonological representations alters the automatization of grapheme-phoneme associations [14, 27]. This phonological processing deficit is believed to alter the ability to identify, store, retrieve and manipulate phonemes, which are necessary for efficient decoding [16]. Three types of tasks assess phonological representations: Phonemic awareness tasks, such as phonemic segmentation, phonological short-term memory tasks, such as non-word repetition [28], and rapid serial naming tasks such as rapid automatized naming [29, 30]. However, rapid automatized naming (RAN) may be considered as “a microcosm of reading” because like reading task it requires fast multimodal integration and several neurological and cognitive processes: Saccadic eye movements, perceptual recognition, visual attention shifts, working memory, lexical access, and articulatory planning [29]. Furthermore, the relationship between reading and RAN does not remain constant throughout development [31]. Numerous studies and meta-analyses have investigated the effects of phonemic awareness training on reading skills. All of these studies agree that phonemic awareness interventions alone are less effective than phonics instruction that combines phonemic awareness and reading fluency trainings [32–34]. However, all of these interventions have only a moderate short-term effect and a small long-term effect on reading [35]. The effectiveness of phonological short-term memory training on reading skills in dyslexic individuals has not been demonstrated in alphabetic languages but has been demonstrated in a logographic language, Chinese [36]. Finally, the results about the training of RAN have yielded mixed results [37]. For example, De Jong and Vrielink [38] did not find effects, Marinus and colleagues [39] did find some effects but these effects did not generalize to reading. Two very recent studies have shown moderate to strong effects of a rapid automatized naming (RAN) training on reading [40, 41]. In addition, some researchers suggest that this deficit in phonological processing is the consequence of a more fundamental deficit in the perceptual and/or attentional processing of auditory information [42–49]. For example, allophonic perception of speech sounds in subphonemic units can lead to poor discrimination of acoustic differences, a perceptual deficit in phoneme categorization and consequently a deficit in phonological processing. Coherently with this view, one study recently showed the positive effect of phoneme categorization training on reading skills and phonemic awareness [50].

According to the visual-attentional hypothesis, a deficit in the visual processing of grapheme representations also alters the automatization of grapheme-phoneme associations. According to this account, the ability to identify, store and retrieve graphemes is disrupted by a deficit in the perceptual and/or attentional processing of visual information. Three types of deficits have been put forward in support of the visual-attentional hypothesis of dyslexia: Perceptual visual deficits [51], deficits in the temporal and spatial displacement of visual-attention [52–55] and elementary visual-attentional deficits [56]. Perceptual visual deficits are related to a hypothetical impairment of the magnocellular visual system, which preferentially processes information with a low spatial frequency and high temporal frequency. Within this theoretical framework, a visual training program called Direction Discrimination Training (DDT) seems to increase reading and phonological processing skills [57]. Regarding visual and spatial attention deficits, the VHSS (Visual Hemisphere Specific Stimulation) program [58], whose goal is to reinforce the inhibitory capacities of the visual-spatial attention processes, seems to be more effective in improving speed and precision reading skills, compared to phonological awareness training [59]. This deficit in the spatial displacement of attention, i.e. of the attentional focus, has also inspired certain remedial activities such as the intensive use of action video games, which has been observed to have a positive effect on reading speed, phonological short-term memory, attentional abilities and crowding in Italian- and also in English-speaking dyslexic readers [60–62]. The results of a systematic review [24] show that visual-attentional interventions based on visual perceptual training and action video games significantly improve reading without explicit phonological or spelling instruction. The third type of visual-attentional deficit concerns the processing of elementary visual-attentional processes. For example, a reduction in the visual-attentional span, that is the number of letters processed simultaneously during eye fixation, appears to disturb the identification of letters and could explain a disorder in the identification of written words [63]. The Maeva© training program has been shown to increase the visual-attentional span, but also improves phonemic awareness and reading of irregular words [50]. Also within the conceptual framework of an elementary visual-attentional deficit, an imbalance between global and local modes of analysis of a complex visual scene can also alter letter encoding, visual identification of irregular words and stabilization of orthographic representation [64, 65]. The Switchipido© program [66] aims at focusing visual attention on global or local levels of analysis to improve reading skills [64, 67].

A third hypothesis considers a potential deficit in cross-modal integration of letters and sounds as a possible cause of the deficit in the automatization of word decoding and consequently of reading fluency. In this theoretical framework, letter-sound association is possible but the simultaneous letter-sound integration as a single automatized audio-visual object is slowed down [3, 68]. This specific deficiency in the link between the processing of orthographic representations and the processing of phonological representations has been found in child and adult dyslexic readers [69, 70] but also in young children at risk of developing dyslexia [68]. The automatic multisensory integration of a single audio-visual object, “the graphoneme” [71] initially appears to activate the left posterior temporal cortical network and then the entire left occipito-temporal-parietal interactive network involved in reading [3, 72, 73]. GraphoGame© [74], a computer-based reading training program, was developed to foster orthography-phonology associations at multiple grain sizes and automatize orthography-phonology integration through the simultaneous and repeated audio-visual presentation of letters, syllables, words, and sentences in a highly playful interface. Developed in Finland the program has been adapted for French [75] and other languages [76–80]. A meta-analysis found positive effects of intervention using *GraphoGame* (GG) if the program was accompanied by a tutor [81]. The French version of GG is broader and more sophisticated than the research versions included in the meta-analysis. A recent validation study of the French version of GG showed effect sizes of around 0.28 for independent practice with the program [82]. Other intervention programs using non-linguistic audio-visual stimuli also show effects on reading skills [83], but the reasons for the deficit in the audio-visual integration of low-level stimuli are still debated [84, 85].

To summarize, independent of the theoretical underpinnings, all intervention programs have attempted to reduce the reading deficit:

- Through interventions on the grapho-phonological and/or orthographic reading processes, by proposing successive training sessions to automatize phonological decoding and lexical recoding [86–88] or by intervening specifically either on the grapho-phonological process or on the orthographic process [89–94];
- Through more specific interventions at the level of an UCD either audio-phonological [95–97], visual [50, 57, 59–61, 98, 99] or audio-visual [83, 100–102];
- Through combined interventions on reading processes and UCD through programs using “phonics instruction” that combine training of phonological cognitive processes and grapho-phonological conversion procedures [103, 104] or through programs that combine training of visual cognitive processes and spelling and reading procedures [59, 105–107].

Previous work has mainly focused on the validation of remediation methods based on three theoretical domains that may explain developmental dyslexia: Phonological theory, visual-attentional theory, and cross-modal theory. Each causal theory generates different hypotheses and multimodal UCD. However, the expression of these UCD is also highly variable depending on the dyslexic reader [108]. Some dyslexic children show the predicted deficit and others do not. Similarly, intervention programs seem to help some readers but not others. Developmental dyslexia may therefore be the consequence of several UCDs [16] that need to be identified in a first step and treated in a second step in order to construct and automatize optimal grapheme-phoneme associations in a third step [5]. Furthermore, it appears that remediation programs that combine an intervention on both UCD and reading processes may be more effective than programs that intervene only at the level of UCD [33, 34].

**Fig. 1:**
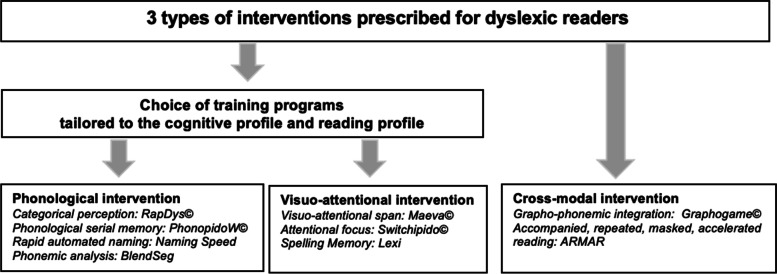
Schematic description of the three intervention programs and the individually tailored training programs

The originality of our study protocol is to combine three types of intervention programs that target phonological, visual-attentional and cross-modal UCD using a multimodal remediation approach in a longitudinal study. All participants benefit from the three types of training: Phonological, visual-attentional and cross-modal. For each participant, an initial assessment of the underlying cognitive processes determines a cognitive profile. Similarly, an assessment of reading and spelling skills will determine a reading profile. The cognitive profile and the reading profile of each dyslexic reader defines the specific type of intervention in each domain (e.g., a child can receive 4 different phonological interventions as a function of the profile, see Fig. 1). Thus, each intervention program focuses on both an underlying cognitive process (visual-attentional, phonological or cross-modal) and a reading process (decoding and orthographic processing). Interventions are therefore individualized, systematic, intensive and evidence-based in accordance with DSM-5 guidelines. Furthermore, daily exercises are performed at home on computerized devices in accordance with the general principles of cognitive rehabilitation [109] and the principles of efficiency and generalization of an intervention [88]. Computerized training programs are adapted to each child’s abilities, progression and cognitive functioning. Computerized programs allow home training, ensure reproducible multimodal presentation and increase autonomy, motivation and attention [110]. Each computer-based training program targets a phonological, visual-attentional or cross-modal cognitive process and is systematically associated with reading and writing exercises of words or pseudowords to promote multisensory learning [111, 112]. Carried out in a therapeutic context, this longitudinal prospective study allows for an immediate clinical application.

## Objectives {7}

### Main objective

The main objective of this study is to assess whether the combination of three types of intensive interventions (phase 2) improve reading and spelling skills compared to baseline (i.e., non-intensive intervention, phase 1).

### Secondary objectives

1. Assess the efficiency of each intervention against baseline.
2. Assess the efficiency of each intervention as a function of the severity of the “underlying cognitive deficits”.
3. Assess the effects of intensive intervention (phase 2) compared to baseline (phase 1) on reading interest, academic performance, and self-esteem.
4. Assess the stability of the intervention effects in all of the above measures 2 months after training.

## Trial design {8}

The trial is a two-arm crossover multicenter randomized controlled trial conducted in France with 76 speech-language therapists as associate investigators. Randomization will be ensured by means of block randomization with a 1/1 allocation. The framework of this trial corresponds to its overall objective which is to test the superiority of a multimodal and intensive training compared with a non-specific and non-intensive training. Indeed, all children who come to seek dyslexia therapy with a speech and language therapist deserve to be helped and it is ethically difficult to justify that we postpone a potentially effective intervention for 16 months (i.e., children would lose more than an entire year, which is a lot during such a critical developmental phase). For these ethical reasons, we have decided to not to include a no-training control group.

**Fig. 2:**
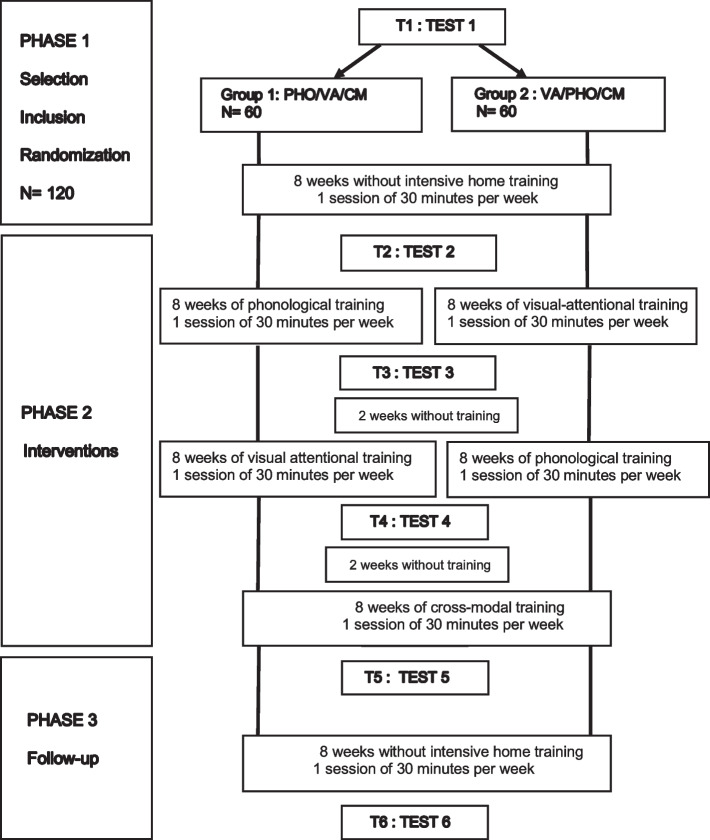
Schematic description of the trial design (see text for details)

The experiment will take place in three phases over 16 months (see Fig. 2):

- Phase 1 (baseline): All dyslexic readers participate in one 30-minute session per week with the speech-language therapist for 8 weeks without intensive home training. Reading and spelling exercises are performed as part of the speech and language therapy.
- Phase 2 (intervention): All dyslexics receive three types of intensive interventions, phonological (PHO), visual-attentional (VA), and cross-modal (CM), that last 2 month each. The order of the first two interventions (PHO and VA) is swapped in two randomly assigned groups of 60 dyslexics each. All children also participate in one 30-minute session per week with the speech-language therapist. For 15 minutes, the speech-language therapist checks that the at-home training instructions are clearly understood. The rest of the session is focused on reading and spelling exercises.

CM training takes place at the end of the protocol for two reasons. The first reason is that it might be necessary to first focus on the phonological and visual-attentional processes per se. PHO and VA training should initially improve orthographic and phonological processes and cross-modal training should subsequently re-establish the link between the processing of orthographic representations and the processing of phonological representations. The second reason is that the full randomization of the order of the three types of training would require a much larger sample and more groups. This would increase the risk of not reaching the needed number of inclusions. The number of participants in each group would then be insufficient to obtain sufficient statistical power.

Each training phase lasts for 8 weeks, 15 minutes per day and 5 days per week. Each child benefits from a program adapted to the specific phonological and visual-attentional deficits identified by the cognitive and language profile assessment. For example, children with phonological deficits may receive training to improve phonological awareness and/or phonological short-term memory and/or rapid automatized naming and/or categorical perception (see more explanations p.18). Children with visual-attentional deficits may receive training to improve global-local analysis and/or visual-attentional span. Training will focus on the most impaired deficits. The CM training is the same for all children.

- Phase 3: All children have one 30-minute session per week with the speech-language therapist over a period of 8 weeks without intensive home training as in Phase 1.

### Main hypothesis

Intensive and specific multimodal training significantly improves reading (fluency, comprehension) and spelling skills compared to a conventional non-intensive and non-specific therapy. These effects of intervention will be tracked after the three training phases (test 5) and 2 months later (test 6).

### Secondary hypotheses

- *Hypothesis 1*: Intensive phonological or visual-attentional training is more effective than conventional non-intensive and non-specific therapy
- *Hypothesis 2*: Intensive, specific, multimodal training (test 5) significantly improves reading interest, academic performance, and self-esteem compared to conventional non-intensive therapy (test 2).
- *Hypothesis 4*: These intervention benefits should last 2 months later (test 6).
- *Hypothesis 5*: No significant difference as for the effects on reading skills is expected between phonological versus visual-attentional training (test 3).

## Methods: participants, interventions and outcomes

### Participants

### Study setting {9}

Study participants are selected and included by associate investigators in their respective centers located in several cities in France. The list of investigators is kept at the Fondation LENVAL Pediatric Hospitals of Nice CHU-Lenval, Delegation for Clinical Research and Innovation (DCRI) of Nice University Hospital Center, and can be requested from the clinical research associate.

### Eligibility criteria {10}

#### Criteria for inclusion

- Age ≥ 8 years old and ≤ 13 years old.
- Diagnosis of a specific neurodevelopmental reading disorder, according to DSM-5 criteria, with scores ≤ − 1.5 standard deviation from the mean on leximetric tests: Alouette [113, 114], Evaléo 6–15© [115] or Evalec© [116].
- Phonological deficit: Scores in speed or accuracy lower than 1.5 SD from the mean for phonemic analysis tasks or non-word repetition tasks or automatized quick naming tasks of the Evalec Battery.
- Visual-attentional deficit: Scores in speed or accuracy lower than 1.5 SD from the average for global or partial reporting tasks of the Evadys© test [117] or for attentional focus tasks of the Sigl© test [118].
- All children have both at least one phonological deficit and at least one visual-attentional deficit.
- Family home equipped with a connected computer set up for daily training sessions.
- Informed consent signed by both parents or by a representative of parental authority.

#### Criteria for non-inclusion

- Intellectual retardation, neurological disorders, pervasive developmental disorder (DSM-5).
- Primary sensory deficit.
- Educational deficiencies, that refer to inadequate schooling (e.g., prolonged or repeated absences from school) or inappropriate teaching (e.g., a teacher who does not follow national guidelines for reading instruction).
- Attention-Deficit Hyperactivity Disorder (ADHD), Specific Language Impairment (SLI).
- Prior intensive daily phonological or visual-attentional training.

#### Exclusion criteria (or study exit criteria)

Participants are temporarily or permanently excluded from the study if:

- They interrupt daily training for more than a continuous period of 15 days without a valid reason determined at the investigator’s discretion.
- They do not participate regularly in daily training, as left to the investigator’s appreciation.
- The participants or their parents withdraw their consent.

The interruption may occur after consent has been obtained or after randomization. A child who wishes to withdraw consent may tell his/her parents or the speech-language pathologist who will inform the principal investigator. Parents may also inform the principal investigator directly (contact information is available at the end of the information sheet that goes with the consent form). For, all patients, the speech-language pathologist must send a patient identification form to the principal investigator indicating the date the consent was signed, the date of randomization, the randomization group, and, if applicable, the date of discharge from the trial.

### Who will take informed consent? {26a}

Oral and written information is given to the parents and the child. Consent form signed by both parents or by legal guardians is taken by each investigator at the site of inclusion.

### Additional consent provisions for collection and use of participant data and biological specimens {26b}

- The processing of data complies with the French Data Protection Act No. 78–17 of January 6, 1978 as amended and Regulation (EU) 2016/679 of the European Parliament and of the Council of April 27, 2016 applicable as of May 25, 2018 (General Regulation on Data Protection, RGPD).
- Data is collected and used in accordance with the reference methodology for processing personal data in the context of health research requiring the express or written consent of the person concerned (MR-001) and has been declared to the CNIL (deliberation no. 2018–153 of May 3, 2018).

## Interventions

### Explanation for the choice of comparators {6b}

#### Main evaluation criteria

Reading efficiency is measured at inclusion (Test 1), at the end of phase 1 without intensive training (Test 2), at the end of the three training periods of phase 2 (Test 5) and after phase 3, i.e., 2 months after the training has stopped (Test 6) by five reading tests:

- Alouette©, a widely used standardized French reading test, measures speed and accuracy when reading a meaningless text and provides a reading age [113] and both speed and accuracy scores [114].
- DeltaText© measures speed and accuracy of meaningless texts matched in number of words, length and complexity [119].
- Mouette-Pingouin© measures the speed and accuracy of word identification of meaningful texts matched in length and complexity to control for a test/re-test effect using two texts controlled for word number and word frequency [115].
- Eval2M© measures the speed and accuracy of identification of words presented in columns [115].
- Evalec© measures the respective speed and accuracy of identification of regular words, irregular words and pseudowords [116].

#### Secondary evaluation criteria

Related to the secondary objectives:

- Reading efficiency after the phonology training and after visual-attentional training will be measured by the five reading tests in Test 3 for the two groups (G1: PHO/VA/CM, G2: VA/PHO/CM).
- Reading efficiency after the two phonological and visual-attentional training periods conducted in a different chronological order in each group, will be measured by the five reading tests in T4 for the two groups.
- Spelling skills will be measured by Chronosdictées© [120] which proposes two versions with sentences matched in length, lexical frequency and phonetic complexity.
- Reading comprehension will be measured by the closure test ORLEC L3© [121, 122].
- Reading disorder impact (reading interest, academic performance and self-esteem) will be measured by two questionnaires completed by each child and his or her parents, in which they express their degree of agreement or disagreement.

### Intervention description {11a}

Each intervention lasts 8 weeks, 15 minutes a day, 5 days a week and combines training for an underlying cognitive deficit (UCD) with training of a reading process. Phonological and visual-attentional UCD trainings are associated with word reading and spelling tasks. Cross-modal training is associated with text reading tasks. Within the phonological and the visual-attentional training periods, the precise nature of the training exercises is determined by the individual profile according to the initial evaluation related to Test 1. Cross-modal training is the same for all participants but is adapted to the reading level of each child.

#### Description of phonological training

Training can focus on three underlying phonological cognitive processes: Categorical perception (CP), phonological serial memory (PSM) and rapid automatized naming (RAN). Training only targets the participant’s most deficient phonological processes according to phonological tests in Test 1 and to baselines measured at the beginning of each training (CP, PSM, RAN). Thus, training for each phonological UCD varies as a function of the nature and intensity of each child’s disorder. For example, training may target CP and phonics instruction in the first month and focus on PSM and phonics instruction in the second month for children without RAN deficit. Phonological cognitive deficits training (CP and/or PSM and/or RAN) is always associated with phonics instruction training.

##### Categorical perception training: *RapDys*©

The intervention is implemented on a computerized device using RapDys© software [123]. It aims at modifying an allophonic mode of perception, i.e., an over-discrimination of infraphonemic acoustic differences, into a categorical or phonemic mode of perception. The child learns to discriminate and/or identify increasingly fine acoustic differences between two different phonemes. The Voice Onset Time (VOT) changes from allophonic peaks at +/− 75 ms VOT to phonemic peaks at +/− 5 ms VOT.

The child’s allophonic discrimination skills are assessed by a discrimination task and a syllable identification task (/də/ and /tə/) provided by the software. Depending on the child’s initial performance and response to training, the software progressively reduces the acoustic distance between two phonemes around the phonemic voicing boundary. Training consists in presenting the syllables /də/ and /tə/ on a VOT continuum whose values vary between − 75 and + 75 ms. The frequency transitions F1, F2, and F3 are 200, 2200, and 3100 Hz respectively, and the equilibrium position is 500, 1500, and 2500 Hz respectively. The frequency F0 is constant at 120 Hz. Each syllable of the continuum lasts 200 ms. When the child obtains a score of correct responses in discrimination or syllable identification that stabilizes above 75%, the next step is implemented.

##### Phonological serial memory training: *PhonopidoW*©

When the child’s score on the non-word repetition subtest indicates deficient performance of phonological short-term memory [116], training with PhonopidoW© software [124] is proposed. This intervention consists in exercises that require discriminating one sound among a sequence of several sounds, comparing or composing sequences of sounds. Contrary to the categorical perception task, the trained phonemes do not present any frequency variations. The phonological proximity between the sounds and the phonological memory load can be parameterized. Each syllable heard is simultaneously associated with a written presentation of the phoneme. Thus, this software also aims at strengthening the grapho-phonological conversion process. The clinician can remotely recommend the specific exercises to be performed.

##### Rapid automatized naming training: *naming speed*©

**Fig. 3:**
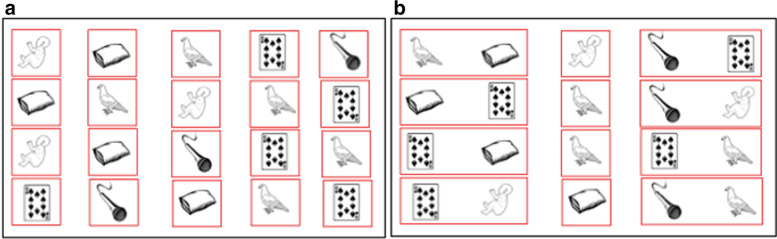
Two examples of screens from the Naming Speed© phonological training program

If the score of the RAN subtest is pathological [116], the digital Naming Speed program inspired by the Italian Run the RAN program [40] is proposed to the child. This program was created specifically for this study since there is no equivalent in French. This computerized intervention trains children to name more rapidly non-alphanumeric visual stimuli and consequently trains the different cognitive processes involved in reading such as left-to-right visual scanning, visual processing complexity, visual attention, lexical access and phonological planning. Five images of black and white objects from the LEAD lexicon database [125] are presented on the screen and randomly repeated on horizontal lines in matrices of 20 to 60 stimuli (see Fig. 3). The child names the images, in the reading direction, keeping the naming speed provided automatically by a red cursor around the image to be named. As the training progresses, the naming speed is increased from a single image to 2, 3, 4 and 5 images to be named simultaneously. The required naming speed increases as the red cursor duration per stimulus decreases from 200 ms to 50 ms. The distance and lexical frequency of the stimuli progressively decrease at the same time as the phonological complexity and the number of stimuli increase. The training is completed when the child reaches the naming speed expected for his or her age, according to the French standards of the Evalec© [116] and La BALE tests [126].

##### Phonics instruction training: BlendSeg

First, phoneme segmentation and blending skills are orally trained [127]. The task of phoneme blending into 10 words or pseudowords requires the child to blend orally several phonemes (e.g., */k/a/t/: cat*). The segmentation task is the reverse activity: 10 words or pseudowords are spoken and then segmented into phonemes by the child (e.g., *dog: /d/o/g/).* Second, the blended and segmented words and pseudowords are then read and written by the child. After copying them, the child is asked to write down the words she or he remembers.

#### Description of visual-attentional training

Similarly to phonological training, only the deficient processes identified in test 1 of Evadys© [117] and Sigl© [118] tests are trained. Visual-attentional cognitive deficits training (visual-attentional span and/or global/local analysis) is always associated with spelling memory training. The duration of the training of each visual-attentional process varies according to the nature and intensity of the disorders. For example, training may target visual-attentionnal span (Maeva©) and spelling memory (Lexi©) in the first month and global/local analysis (Switchipido©) and spelling memory (Lexi©) in the second month for children with two deficits.

##### Visual-attentional span training: *Maeva©*

**Fig. 4:**
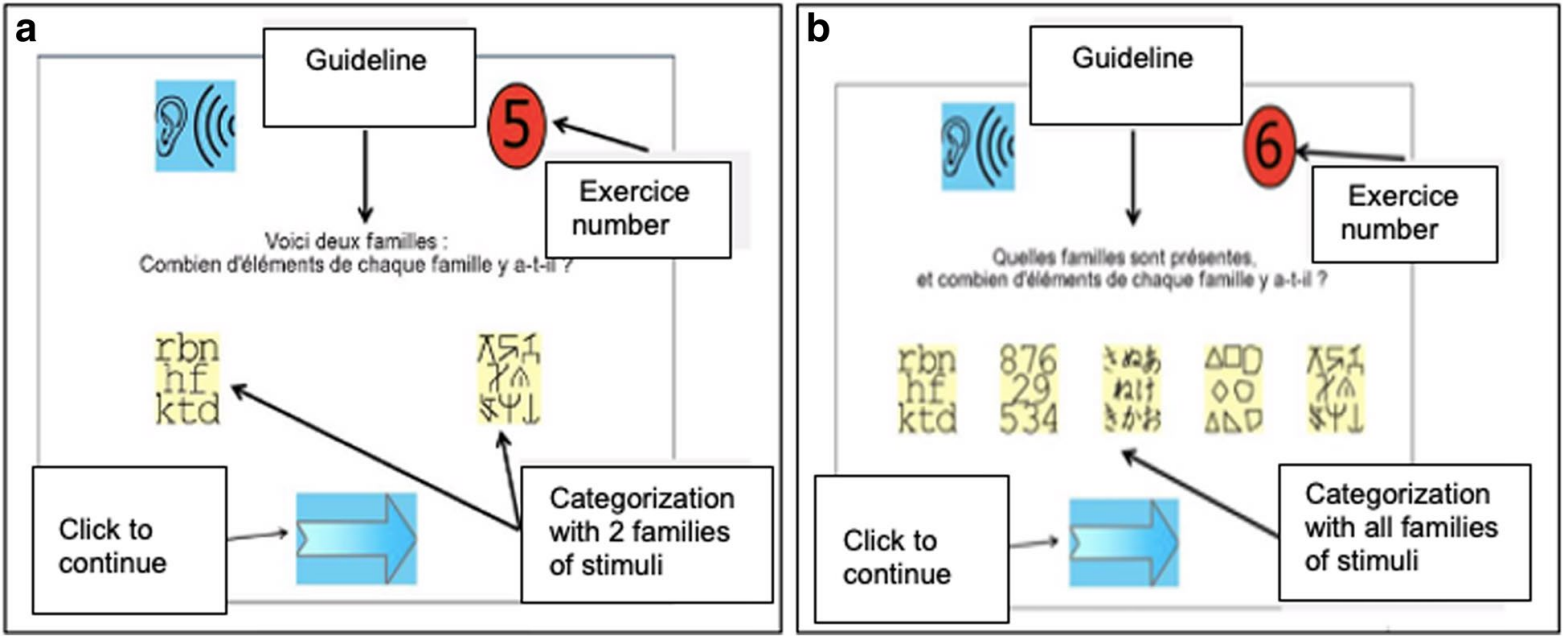
Two examples of the Maeva© visual-attentional training program: (**a**) categorization with two families of stimuli, (**b**) categorization with all families of stimuli

 This program [128] is designed to increase the visual-attentional span, i.e. the number of visual elements to be processed simultaneously during ocular fixation. In these exercises, a sequence of two to seven visual stimuli including letters, pseudo-letters, numbers, geometric shapes or Japanese characters is briefly (420 to 120 ms) presented on the screen. When the sequence disappears, the child must perform a categorization task following six different instructions of increasing difficulty (e.g., Fig. 4). An algorithm adapts the difficulty of the exercise in real time, taking into account the child’s previous responses to keep the child’s success rate around 75%.

##### Global vs. local analysis training: *Switchipido*©

**Fig. 5:**
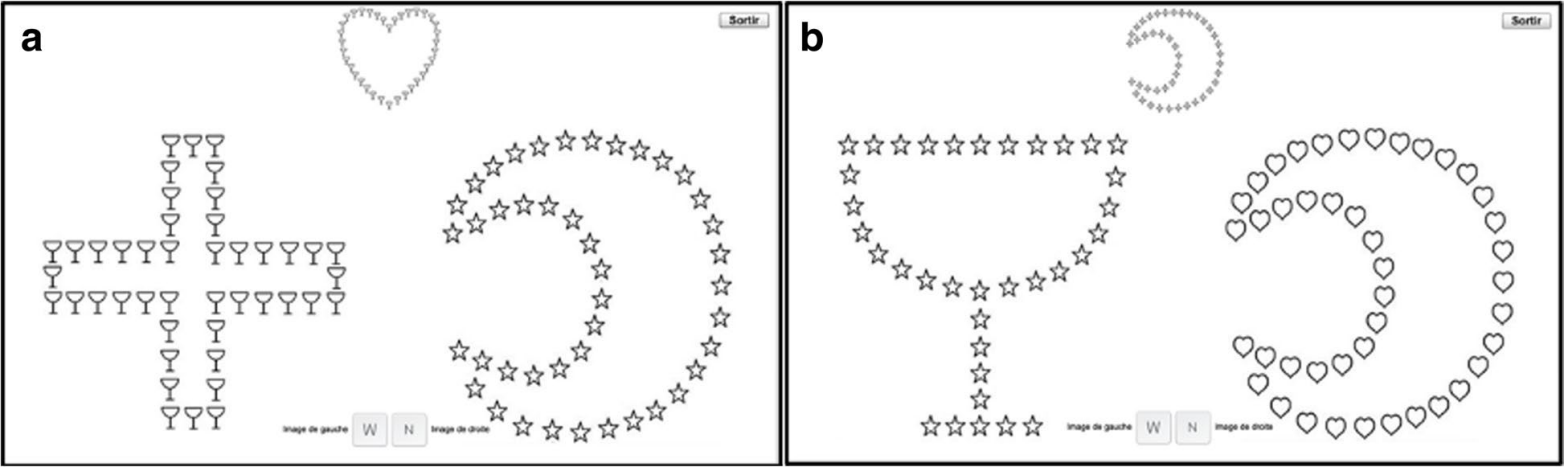
Two examples of the Switchipido© visual-attentional training program: (**a**) focus on the global level, (**b**) focus shift between the local and global level

This program, also computerized, is based on the Switchipido© software [66] which stimulates the visual-attentional switch between global and local levels of information processing. The display of hierarchical stimuli (large stimuli made of small drawings) allows a visual focus on the global shape and mobilizes the visual focus shift between the global and local levels (e.g., Fig. 5). The aim of these exercises is to reinforce the mechanisms of voluntary inhibition of details and the spontaneity in the global processing of complex visual stimuli.

##### Spelling memory training: *Lexi*©

This program, which is based on the principle of a mnemonic encoding/storage/retrieval process [129] consists of a “flash” reading of 20 semantically known words. After being read aloud by the child, the written words are presented again for a longer but limited time so that the child can copy them manually. After copying them, the child is asked to write down the words she or he remembers. The word lists are created by the speech-language therapist and adapted to each child according to his or her school level, spelling level and spelling regularity. In the phonological training, blending and segmentation is trained orally with 20 words or pseudowords. The same words are then read and written. In the visual-attentional training, the 20 words are also read and written. So, the amount of reading and spelling is similar in both training conditions.

#### Description of cross-modal process training

The phonological and visual-attentional training methods described above are based on either the auditory or non-simultaneous visual presentation of linguistic or non-linguistic units.

Cross-modal training is based on the simultaneous and repeated bimodal audio-visual presentation of linguistic units.

##### Grapho-phonemic integration training: GraphoGame©

**Fig. 6:**
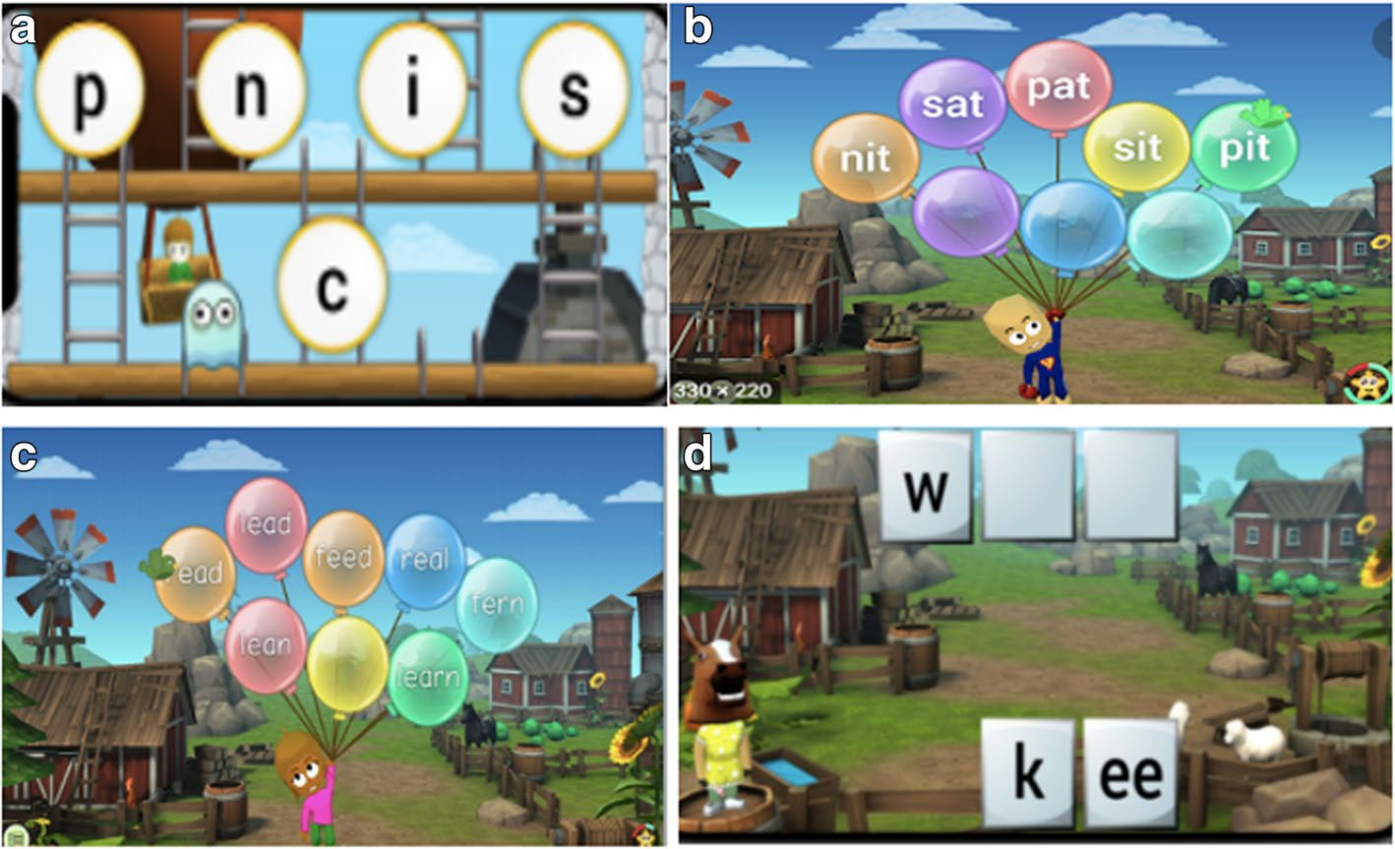
Four examples of the GraphoGame© cross modal training program. The child hears and chooses one of the proposed options: (**a**) the phoneme /c/, (**b**) the syllable /sat/, (**c**) the word “real”, (**d**) the child hears the word “week” and must put the letters in the right order

The objective of this program is to reinforce the coupling of the orthographic code with the phonological code by reinforcing the specific cross-modal (i.e., audio-visual) link between letters and sounds. GraphoGame© software [74] is an audio-visual training program that simultaneously presents auditory and written stimuli for linguistic units of different sizes (phonemes, syllables, rhymes, words and sentences). This software, designed as a serious game, offers the child a game progression that becomes more and more complex. After listening to an oral stimulus, the child must click on the corresponding written presentation chosen from among several written stimuli (see Fig. 6). Twenty eight sequences are proposed, and each sequence offers 6 to 12 levels. If the success rate is less than 85%, the child must repeat the level.

##### Reading fluency training: Accompanied, Repeated, Masked, Accelerated Reading (ARMAR)

This training consists in stimulating a child’s reading fluency and interest for reading [130, 131]. Each day, the child chooses a text from a book adapted to his/her reading level and must read the text in 6 steps. (1) The child reads the text aloud for 2 minutes. The parent notes the number of errors. (2) The parent reads the same text aloud at normal speed while the child follows the text that is read with a cursor (binary audio-visual processing). (3) Parent and child read aloud at the same time (accompanied reading) and the child follows the text with the cursor. (4) The child reads alone and again follows the text read aloud with the cursor; the parent corrects the wrong words or reads the words that are not read fast enough (repeated reading). (5) The child reads the text aloud alone, as quickly as possible, while listening to music of his or her choice with headphones (auditory masking). (6) The child reads the text aloud alone, as quickly as possible, without masked hearing. The parent notes the reading speed and the number of errors. The next day, a different text is read following the same steps.

### Criteria for discontinuing or modifying allocated interventions {11b}

The criteria for discontinuing or modifying allocated interventions are: (a) a serious adverse event (disease or pandemic) or environmental event (relocation), (b) withdrawal of consent, (c) cessation of daily training for more than 15 days without valid reason and subject to the judgment of the investigator, or an irregularity in daily training subject to the judgment of the investigator. Post-intervention measures for children that drop out from the intervention will be taken to perform an intention-to-treat analysis.

### Strategies to improve adherence to interventions {11c}

The recordings of the sessions carried out at home on the digital platforms of the various software programs that make up this protocol allow for precise control of the date, duration, frequency and scores of each training session. Weekly face-to-face visits with the speech-language therapist serve to longitudinally control compliance with the intervention. Protocol monitoring sheets indicating the number of sessions per week are filled out by the parents after each training session (see “parent sheets” in Additional files 2, 3, 4, 5).

### Relevant concomitant care permitted or prohibited during the trial {11d}

Weekly orthoptic and/or psychological care are not prohibited. Participation in another program of intensive care for a learning disability is a criterion for exclusion.

### Provisions for post-trial care {30}

This protocol is part of an ongoing speech and language therapy treatment. Its objective is to propose an intervention model that can be used in the treatment of a written word identification disorder. This type of evidence-based intervention is therefore a benefit for the patient who will be able to continue his or her care with the therapist who, in turn, will have strengthened his or her care practices after participating in this study.

### Outcomes {12}

The results of the study will be published 16 months after the last inclusion. Inclusions continue until September 2021.

### Participant timeline {13}

The schedule of enrolments, interventions and evaluations is presented in Table 1.

**Table 1:**
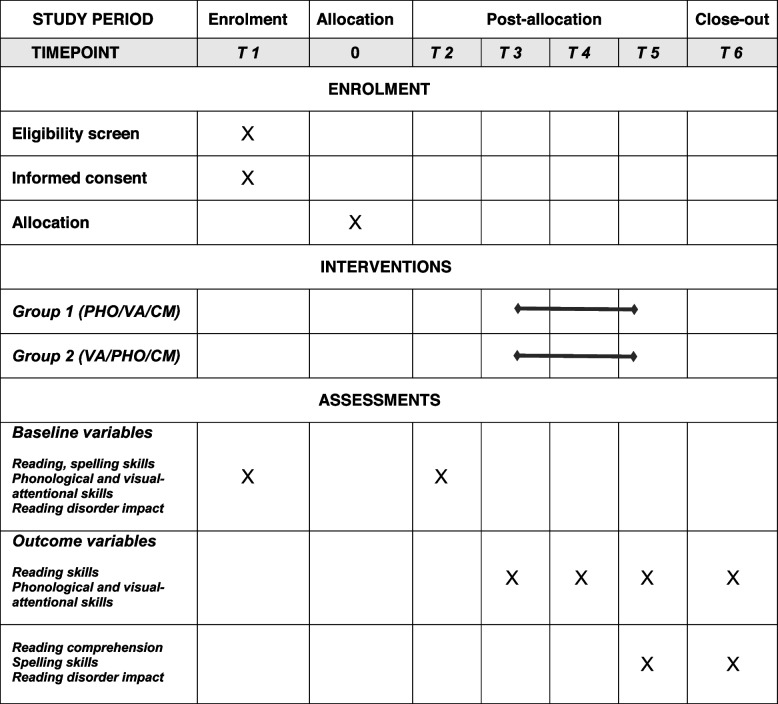
Participant timeline

### Sample size {14}

In a pilot study on 20 patients with dyslexia, we determined that the effect size on reading efficiency between classical rehabilitation (baseline) and intensive rehabilitation was Cohen d = 0.867. Given that the literature on interventions for children and adolescents with reading disabilities gives effect sizes of 0.322 for phonological interventions (see [34]), expected effects on reading skills of a multimodal phonological, visual-attentional and cross-modal intervention should be greater. However, we preferred to be conservative and reduced the expected effect size to Cohen’s d = 0.700. Using G*Power 3.1.9.6, we found *n* = 55 per group. With an estimated drop-out rate of 10%, we will therefore include 120 participants.

### Recruitment {15}

The study is multicenter: 76 associate investigators have been recruited in France through the *Centre Référent des Troubles des Apprentissages* (CERTA) at the Nice-Lenval University Hospital Center and through professional training organizations. Each associate investigator may include a number of participants not defined in advance. If indeed associate investigators (i.e., speech and language therapists) include more than one patient, we will add the multi-level structure to the statistical data analyses. All the investigators recruited are state-qualified speech-language therapists and have expert practice in using these assessment and training methods.

## Assignment of interventions: allocation and blinding

### Allocation

### Sequence generation {16a}

Randomization is performed centrally at the Delegation for Clinical Research and Innovation of Nice (DRCI) of the Nice University Hospital Center by e-mail. Randomization is not stratified on the participating centers; the recruitment is competitive.

### Concealment mechanism {16b}

Randomization lists are produced using Query Advisor® v 7.0 software.

### Implementation {16c}

The DRCI assigns participants to interventions. The participant’s treatment group and inclusion number are then relayed to the investigator.

### Blinding

### Who will be blinded {17a}

Not applicable.

### Procedure for unblinding if needed {17b}

The clinical trial is open-label randomized since the intervention programs must be known by the investigators and the participants before the beginning of the experiment to ensure that the training runs smoothly.

## Data collection and management

### Plans for assessment and collection of outcomes {18a}

The assessment plan and all the data collected are entered in three Case Report Forms (CRFs) available online: The CRF Test 1 indicates the initial assessment plan and allows for baseline data collection. Group 1 and Group 2 CRFs indicate the assessment plans for each arm and allow for the collection of data in T2, T3, T4, T5 and T6 (see Additional files 6 and 7). Data quality is optimized by computerized assessments for all tests except for two leximetry tests (Alouette© and DeltaText©), a reading comprehension test (Orlec© L3), a spelling test (Chronosdictées©) and a self-esteem questionnaire. All the tests selected are referenced and scientifically validated (see Table 2).

**Table 2:** An overview on the assessment batteries

Cognitive Processes		Measures	Labels
**Reading and spelling assessments**	Reading aloud	Meaningless texts reading	Alouette© /DeltaText©
		Meaningful texts reading	Evaléo©
		2 min word reading	Evaléo©: Eval2M
		Regular, irregular, pseudo-words reading	Evalec©
	Reading comprehension	Multiple choice statements	Orlec 3©
	Spelling	Phonetic, lexical and grammatical spelling	Chronosdictées©
**Underlying cognitive processes assessments**	Phonological process	Phonological analysis	Evalec©
		Phonological short-term memory	Evalec©
		Rapid automatized naming	Evalec©
		Categorical perception	RapDys©
	Visual-attentional process	Visual-attentional span	Evadys©
		Global/local analysis	Sigl©
**Complementary assessments**	Span memory	Digit span	Evaléo©
		Visual-spatial span	Corsi©
	Reading disability impact	Reading motivation, academic performance and self-esteem	Likert scales
	Oral language	Vocabulary and syntactic comprehension	Evaléo©
	Matrix Reasoning	Fluent and visual-spatial intelligence	Wisc 5©

#### Word identification assessment

##### Meaningless texts

*Alouette*© [113, 114]: This test (265 words) which is considered the gold standard of leximetry tests assesses speed and accuracy when reading a meaningless text. This test provides a reading age [113], a speed score, a precision score, a precision index and a speed index [114]. The text includes trick questions for readers who tend to massively use contextual anticipations (“*poisson*” instead of “*poison*” after “*lac*”) or identification behaviors (items that are visually or phonologically close, such as “*amie / annie*” or “*gai / geai*”). The text is surrounded by drawings that induce contextual errors (drawing of a “*écureuil*” near the word “*écueil*”). Time limit for reading is 3 minutes. 

*DeltaText©* [119, 123]: Four different meaningless texts whose words (*n* = 201 words) are balanced in length, lexical frequency, and syllabic and phonemic complexity are proposed to limit a possible test/re-test effect. All words are regular to allow reading even if the spelling lexicon is insufficient. Time limit for reading is 3 minutes. The number of errors and the reading time are recorded.

##### Meaningful texts

*Evaléo*©, *Mouette/Pingouin* [115]: These two meaningful texts (*n* = 450 words) are balanced in word and sentence length, lexical frequency and syllabic and phonemic complexity in order to limit a re-test effect. Reading time and accuracy are recorded. Time limit for reading is 3 minutes.

##### Identification of written words

*Evaléo*©, *Eval2M* [115]: This test (*n* = 263 words) assesses the percentage of words read correctly in a limited time of 2 minutes. Words are presented in 10 columns and according to length and frequency. 

*Evalec©* [116]: This test assesses reading of regular words, irregular words and pseudo-words. This computerized test presents the words to be identified one by one on the screen. The particularity of this test is to use voice detection to measure the time needed to correctly read the words. The lexical or sublexical reading processes are assessed by calculating the latency time in ms when correctly reading items and the percentage of errors when reading regular words (*n* = 36), pseudo-words (n = 36) and irregular words (n = 36). The length effect is assessed by comparing the accuracy and speed measures for short and long irregular words (*n* = 20) with those for short and long pseudowords (n = 20).

#### Reading comprehension assessment

The *ORLEC L3©* [121, 122] assesses word decoding speed and sentence comprehension. This closure test presents sentences to be completed (n = 36) with a word selected among 5 proposed words. The raw score corresponds to the number of correct items completed in 5 minutes.

#### Spelling assessment

*Chronosdictées*© [120] assesses lexical, morphosyntactic and phonetic spelling. Two dictations, A and B, of sentences are used for each grade level in elementary and middle school. The results are expressed in number of errors: 3 scores for the number of phonetic, lexical and grammatical errors, 2 scores for segmentation errors and omissions of words and a score for the total number of errors.

#### Assessment of underlying cognitive processes

##### Categorical perception assessment

Allophonic discrimination skills are assessed by syllable identification and discrimination tasks (/də/ and /tə/) using RapDys© software [123]. The tasks consist in discriminating and identifying increasingly fine acoustic differences between two different phonemes. The Voice Onset Time (VOT) changes from allophonic peaks at +/− 75 ms VOT to phonemic peaks at +/− 5 ms VOT.

##### Phonological processes assessment

For the following tests, Evalec© software calculates a score for precision and for speed [116].

- The pseudoword repetition task assesses phonological short-term memory and is composed of pseudowords (*n* = 12) with a simple syllabic structure and pseudowords (*n* = 12) with a complex syllabic structure, both with 3 to 6 syllables.
- The task of deleting the first syllable of trisyllabic pseudowords (*n* = 10) assesses the phonological analysis (e.g., povidu/vidu). The two tasks of deleting the first phoneme of monosyllabic pseudowords (*n* = 24) assess the phonemic analysis (e.g., puf/uf and pra/ra).
- The color-naming task assesses rapid automatized naming. A matrix of color images (*n* = 54) and a matrix of written color names (*n* = 54) are presented in 9 lines of 6 colors in random order. Three color names have a CVC syllabic structure *(rouge, jaune, vert)* and three have a CCV syllabic structure *(bleu, blanc, gris).*

##### Visual-attentional processes assessment

Visual-attentional span is measured by global and partial letter report tasks with Evadys© software [117]. In the Global Report condition, the participant must name a sequence of five consonants presented for 200 ms. In the Partial Report condition, a vertical bar appears and indicates the position of the letter to be named among the five letters presented for 200 ms. The letter sequences are formed so as not to activate any memorized lexical knowledge and so as to minimize crowding. Beforehand, an isolated letter identification task is presented in order to control for single letter processing speed. The software calculates a score based on the number of successful sequences and a letter span. 

Sigl© software [118] assesses the ability to focus attention on a global or local mode of analysis of visual information. The stimuli are drawings of hierarchical objects presented for 175 ms for which a global or local level is selected as per the instruction of a target. The software calculates the difference in performance between Control and Interference conditions to assess the local and global interference in response time and percentage of errors. To calculate the asymmetry of the interference, the local interference effect is subtracted from the global interference effect and this difference is typically positive.

#### Span memory assessment

Assessment of the digit span with Evaléo© software [115]: Repetition of a series of 2 to 7 digits in forward (short-term memory) and backward (working memory) order assesses verbal memory. The number of digits correctly repeated determines the digit span.

Assessment of the visual-spatial span [132]: The Corsi block test consists in reproducing, in the same or reverse order, the sequence in which the clinician points to different cubes. The number of cubes in the sequence gradually increases, thus determining the visual-spatial span.

#### Assessment of reading disability impact

Changes in reading disability, reading motivation, academic performance and self-esteem are measured by two questionnaires. These questionnaires consist of several statements for which the respondent expresses his or her degree of agreement or disagreement. Two Likert scales are constructed, one for the child, one for the parents, and are proposed before and after training (for more details, see Additional file 8).

#### Optional assessment

If the participant did not have an oral language assessment prior to inclusion, three measures will be taken to assess vocabulary expression, lexical syntactic comprehension. Naming Vocabulary test [115] assesses the lexical stock of known words produced and naming time. The picture/word association [115] assesses the lexical stock. The picture/sentence association [133] assesses syntax understanding in sentences.

If the participant has not had an assessment of intellectual efficiency, the *Matrix Reasoning* test from the Wechsler Intelligence Scale for Children, Wisc-5 [134], is administered to determine an index of fluid and visual-spatial intelligence [135].

### Plans to promote participant retention and complete follow-up {18b}

The principles of therapeutic education and shared decision making are used to maintain the children’s participation during the 16 months. Participants and their parents receive all the information necessary to understand developmental dyslexia and the protocol of care offered to them. This information is presented and repeated throughout the duration of the study on different media (slide show and fact sheets). Most interventions at home are standardized and computerized. The role of the parents is making sure that the children respect the daily sessions, supporting them by being present during the numerical training and carrying out the verbal trainings such as BlendSeg or ARMAR. The children and their parents are also trained in using the software on their own by means of tutorials. The outcomes obtained during the different phases of the study are communicated very precisely and explained to the parents and children. The child can decide to leave the study at any time. The data collected until the withdrawal of consent or the end of participation is kept and analyzed as specified in the information leaflet given to the parents.

### Data management {19}

For each assessment (tests 1 to 6), data are collected in raw scores (number or percentage of errors and completion time in seconds), percentiles or standard deviations. These data are reported on the CRF by each associate investigator, and are checked and centralized on an Excel spreadsheet by the coordinating investigator. In accordance with the Good Clinical Practices of Decree No. 64 of November 30, 2006 (JORF No. 277), at the end of the trial, all documents relating to the protocol are archived for a minimum of 15 years by the principal investigator in a locked room with sufficient guarantees of protection against fire, water damage, light or any malicious acts. Given that all interventions use at least two computerized training programs, treatment fidelity and performance during training will be assessed by analyzing the log files and user data.

### Confidentiality {27}

For the duration of the study, information and data will be collected and archived anonymously by a numeric code randomly assigned to each participant.

Experimental data, collected directly on computer, will be transferred (and deleted from the computer used for the study) to a network secured by an identifier and a password, whose access is limited to the study authors. Experimental data collected in paper format will be entered directly into the observation workbook and will be considered as source data.

### Plans for collection, laboratory evaluation and storage of biological specimens for genetic or molecular analysis in this trial/future use {33}

Not applicable, no samples collected.

## Statistical methods

### Statistical methods for primary and secondary outcomes {20a}

The statistical analysis will first include a descriptive analysis of the study population and the parameters studied, globally and by training group, with an assessment of absolute and relative frequencies (and their 95% confidence intervals) for categorical variables, and an assessment of means and standard deviations, medians and interquartiles for quantitative variables. A flowchart will present the number of eligible participants, the number of participants randomized into each group and the number of participants included in the final analysis. The characteristics of participants lost to follow-up during the study will be described. As recommended in the Consort recommendations, participant characteristics will be compared between the two groups at inclusion from a clinical and statistical perspective. Before each analysis is carried out, the conditions of application of the tests used will be verified. The different tests will be considered significant at the 5% threshold (first species risk).

### Statistical analysis

1. To assess the gains for each dependent variable (reading, spelling and reading comprehension scores) in each group (1 and 2) and for the total sample, we will calculate the means difference and confidence intervals between T1 and T2 (Phase 1 without training), between T2 and T4 (Phase 2 after 16 weeks of training), between T4 and T5 (Phase 2 after 24 weeks of training), and between T5 and T6 (Phase 3 after 8 weeks without training).
2. The gains between T1 and T2 will be compared with the gains between T2 and T4, between T4 and T5, and between T5 and T6 to determine whether the T2T4, T4T5, and T5T6 differences represent a significantly greater gain compared to the no training phase (T1T2).

To measure the effect of each type of training, we will calculate the effect size, *Cohen’s d* or *η*^*2*^, from the gains of each dependent variable. Cohen’s d scores of 0.3, 0.5 and 0.8 are considered to represent small, medium and large effect sizes, respectively.

3. To determine whether the order of PHO/VA versus VA/PHO training leads to different effects, we will perform a repeated Anova with cross-over between groups to compare the gains between T2 and T4 of each group, controlling for gains between T2 and T1 without training. This analysis will also allow us to determine if there is a significant difference between the effects of 8 weeks of phonological training and 8 weeks of visual-attentional training between T2 and T3.
4. Exploratory factor analysis will verify that all the items in the questionnaires (pre-test and post-test Likert scale) or, failing that, some of them can be combined into a score. If necessary, the composite scores will be analyzed as continuous variables. If not, graphical representations in the form of histograms will be used to visualize the variations, and χ^**2**^ tests will be carried out.

### Interim analyses {21b}

The baseline scores obtained monthly during Phase 2 will also be analyzed according to the same statistical strategy presented earlier in 20a.

### Methods for additional analyses (e.g. subgroup analyses) {20b}

Any additional subgroup analysis will depend on the sample sizes of the subgroups analyzed. However, multiple case studies will be considered if necessary.

### Methods in analysis to handle protocol non-adherence and any statistical methods to handle missing data {20c}

Missing data will be replaced by multiple imputed values.

### Plans to give access to the full protocol, participant level-data and statistical code {31c}

Additional data will be available online after recruitment completes.

## Oversight and monitoring

### Composition of the coordinating center and trial steering committee {5d}

Coordination of the clinical trial is guaranteed by the Delegation for Clinical Research and Innovation of Nice University Hospital (DRCI) of the Nice University Hospital Center. Principal Investigator (KHE): Design and conduct of DDMR, preparation of protocol, investigators brochure and Case Report Forms. Scientific Committee (SF, BDC, GL, JZ): Agreement of final protocol, university ethics committee applications, publication of the study reports review. Management Committee (HC, DD): National ethics committee applications, data verification, randomization. Data Manager (JN): Data collection and data entry.

### Composition of the data monitoring committee, its role and reporting structure {21a}

Monitoring of the clinical trial is guaranteed by the Laboratory of Anthropology and Clinical, Cognitive and Social Psychology (LAPCOS) and the Delegation for Clinical Research and Innovation of Nice University Hospital (DRCI) of the Nice University Hospital Center.

### Adverse event reporting and harms {22}

This trial intervention does not imply any adverse event or harms.

### Frequency and plans for auditing trial conduct {23}

The principal investigator is in constant communication with all the associate investigators in the online workgroup.

### Plans for communicating important protocol amendments to relevant parties (e.g. trial participants, ethical committees) {25}

The regional ethics committee (*Comité de Protection des Personnes Ile de France IV*) has approved the trial DDMR protocol (ID no. RCB 2019-A01453–54). The study has been registered in the ClinicalTrials.gov protocol registration system (NCT 04028310). Important protocol modifications will be submitted to the ethical committee CPP IDF VI for validation. Any modifications will be applied and added to the protocol. Modifications will be communicated in a new consent form to trial participants. The new protocol will be transmitted to investigators for application.

## Dissemination plans {31a}

Trial results will be communicated in publications.

## Trial status

Recruitment of participants started in September 2019 and will end in September 2021.

## Supplementary Information

**Additional file 1.** SPIRIT-Checklist (page numbers correspond to each item).

**Additional file 2.** Parent sheet, phonological training: Rapdys, Phonopidow (guidelines and protocol monitoring).

**Additional file 3.** Parent sheet, Phonological training: Rapid Automatized Naming (guidelines and protocol monitoring).

**Additional file 4.** Parent sheet, visuo-attentional training (guidelines and protocol monitoring).

**Additional file 5.** Parent sheet, cross-modal training (guidelines and protocol monitoring).

**Additional file 6.** Case Report Form Test 1 (Assessment and data entry).

**Additional file 7.** Case Report Form Group 1 (Assessment and data entry).

**Additional file 8.** Likert scale-child-parent (Assessment of reading disability impact).

## Data Availability

This manuscript does not contain any data at this stage. The datasets generated and/or analyzed during the current study will be available in supplementary information files or through a permanent weblink to datasets.

## Abbreviations

DDMR: developmental dyslexia and method of remediation
ANOVA: analysis of variance
UCD: underlying cognitive deficits
SD: standard deviation
CP: categorical perception
PSM: phonological serial memory
RAN: rapid automatized naming
CRF: Case Report Form
DRCI: Delegation for Clinical Research and Innovation of Nice
