# Supplementary material for: Multimodal intervention in 8- to 13-year-old French dyslexic readers: Study protocol for a randomized multicenter controlled crossover trial
Source: BMC Pediatr. 2022 Dec 28;22:741. doi: 10.1186/s12887-022-03701-8 (PMC9795620; doi:10.1186/s12887-022-03701-8)
Supplement: Supplementary file 2 — Additional file 2. Parent sheet, phonological training: Rapdys, Phonopidow (guidelines and protocol monitoring). [file 12887_2022_3701_MOESM2_ESM.pdf]

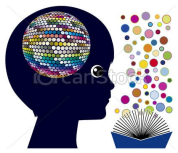

Parent sheet, phonological training: Rapdys, Phonopidow

DDMR Study, 19-HPNCL-02

- Regularly remind your child why he or she needs to do the exercises.
- The speech therapist will tell you which exercises are appropriate for your child's age and difficulties. All you have to do is follow this progression.

Remember that regular practice guarantees effectiveness. So don't replace 2 days (15 min each) with 1 day (30 minutes).

1. Rapdys / Blending: 15 min per day

What for? When we perceive sounds better, we analyze them better and therefore read and write better.

When? For one month, **5 days a week** for **15** minutes:

- 10 minutes of RapDys training with headphones.
- 5 minutes of phoneme blending.

NB: In Rapdys, the child must complete full sessions with a 70% score to move on to the next level. At the end of the session, a message appears: "Send data to the server."

Phoneme blending training is done orally with an adult:

- The child combines the phonemes of 10 words.
- Reads the 10 words (3 times).
- Writes the 10 words (delayed copy or dictation).

Follow-up calendar:

- Mark with an X when training is done.
- Write 0 if the training has not been done.

	Day 1	Day 2	Day 3	Day 4	Day 5
Week 1					
Week 2					
Week 3					
Week 4					

2. Phonopidow / Segmentation: 15 min/day

What for? When we remember the sounds better, we analyze them better and therefore read and write better.

When? For one month, **5 days/week** for 15 minutes:

- 10 minutes of training with Phonopidow.
- 5 minutes of word segmentation.

NB: The progression and type of exercise will be specified by your speech therapist.

Phoneme segmentation training is done orally with an adult:

- The child divides 10 words into phonemes.
- Reads the 10 words (3 times).
- Writes the 10 words (delayed copy or dictation).

Follow-up calendar:

- Mark with an X when training is done.
- Write 0 if the training has not been done.

	Day 1	Day 2	Day 3	Day 4	Day 5
Week 1					
Week 2					
Week 3					
Week 4					

At the end of the training (2 months), bring the completed card back to your speech therapist.

Comments:

- Did your child willingly complete the exercises: yes, no
- What exercises did he or she like?
- What exercises did he or she dislike?
- What problems did you encounter and with what software?
- Other remarks:

First Name :
Name :
Date :
