# Supplementary material for: Multimodal intervention in 8- to 13-year-old French dyslexic readers: Study protocol for a randomized multicenter controlled crossover trial
Source: BMC Pediatr. 2022 Dec 28;22:741. doi: 10.1186/s12887-022-03701-8 (PMC9795620; doi:10.1186/s12887-022-03701-8)
Supplement: Supplementary file 3 — Additional file 3. Parent sheet, Phonological training: Rapid Automatized Naming (guidelines and protocol monitoring). [file 12887_2022_3701_MOESM3_ESM.pdf]

Parent Sheet, Phonological training: RAN or Rapid Automatized Naming

DDMR Study, 19-HPNCL-02

Regularly remind your child why he or she needs to do the exercises.
The speech therapist will tell you which exercises are appropriate for your child's age and difficulties. All you have to do is follow this progression.
Remember that regular practice guarantees effectiveness. So don't replace 2 days (15 min each) with 1 day (30 minutes)

RAN - segmentation: 15 min per day, 5 days per week

What for? By quickly naming pictures in a row from left to right, we train the same mechanisms that are involved in reading, i.e. visual scanning, visual attention, rapid access to a word's phonological representation (how it is pronounced) and semantics (what it means).

When? For one month, **5 days/week** for **15 minutes/day:**

- 10 minutes of RAN training.
- 5 minutes of phoneme segmentation.

RAN training is done with a PowerPoint file. Simply name the pictures as soon as the red square appears:

- If the task is successfully completed (no mistakes and time respected), you can move on to the next slide.
- If the task is not successfully completed (errors or inability to complete it within the allotted time), repeat the slide until full success is achieved.
- Note errors in the table below (write the number of errors and the time taken).
- If unable to succeed, move on to the next exercise.

Phoneme segmentation training is done orally with an adult:

- Divide 10 words into segments.
- Read the 10 words (3 times).
- Write the 10 words (delayed copy or dictation).

Follow-up calendar:

- Mark with an X when training is done.
- Write 0 if the training has not been done.
- Add the number of errors and the time if the task is not successfully completed

	Day 1	Day 2	Day 3	Day 4	Day 5
Week 1					
Week 2					
Week 3					
Week 4					

Remarks:

- Did your child willingly complete the exercises: yes, no?
- What exercises did he or she like?
- What exercises did he or she dislike?
- What problems did you encounter and with what software?
- Other remarks:

First name:
Last name:
Date:
