# Supplementary material for: Multimodal intervention in 8- to 13-year-old French dyslexic readers: Study protocol for a randomized multicenter controlled crossover trial
Source: BMC Pediatr. 2022 Dec 28;22:741. doi: 10.1186/s12887-022-03701-8 (PMC9795620; doi:10.1186/s12887-022-03701-8)
Supplement: Supplementary file 4 — Additional file 4. Parent sheet, visuo-attentional training (guidelines and protocol monitoring). [file 12887_2022_3701_MOESM4_ESM.pdf]

Parent sheet, visuo-attentional training

DDMR Study, 19-HPNCL-02

- Regularly remind your child why he or she needs to do the exercises.
- The speech therapist will tell you which exercises are appropriate for your child's age and difficulties. All you have to do is follow this progression.

Remember that regular practice guarantees effectiveness. So don't replace 2 days (15 min each) with 1 day (30 minutes).

1. Maeva /Lexical Access: 15 min/day

What for? When we see more letters at the same time, we analyze them better and therefore read faster and write better.

When? For one month, **5 days/week** for **15 minutes/day:**

- 10 minutes of training with Maeva.
- 5 minutes of spelling memory training (Lexical Access in PowerPoint).

Spelling memory training is done with an adult:

- The child reads the words: if the child makes a mistake, the parent should read the word correctly aloud.
- Explain the meaning of unknown words.
- The child writes the words while the slides scroll at normal speed.
- Tell the child to write at his own pace, even if he or she can’t write all the words.
- At the end, go back to each slide and check that the word is written and that it is correct.
- If there is an error, the child writes the word after looking only once at the screen: the child looks at the word and writes it completely without looking at the screen again.

NB: Repeat the same list of words for 3 consecutive days and then change list when there are no more errors (words and progression have to be adapted to the child's difficulties).

Follow-up calendar:

- Mark with an X when the training is done and successfully completed.
- Otherwise put the number of words correctly written in the box.
- Put 0 if the training has not been done.

	Day 1	Day 2	Day 3	Day 4	Day 5
Week 1					
Week 2					
Week 3					
Week 4					

2. Switchipido /Lexical Access: 15 min per day

What for? When we pay more attention to words and letters, we see them faster and therefore read faster and write better

When? For one month, 5 **days a week** for **15 minutes:**
- 10 minutes of training with Switchipido.
- 5 minutes of spelling memory in the same way as the first month

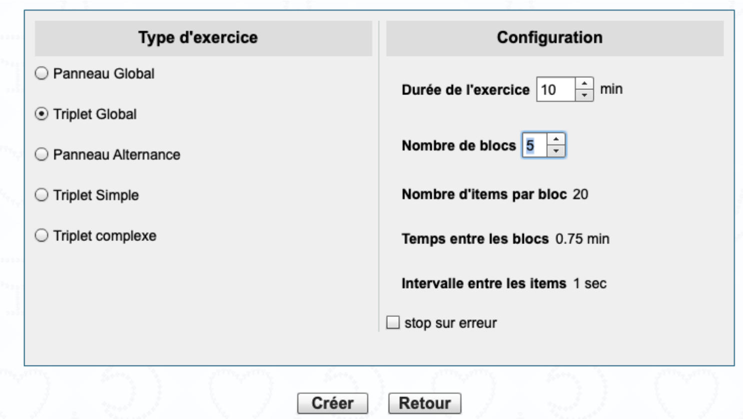

Duration of exercise: 10 min
Number of blocks: maximum

Example of progression:
Week 1: Global Panel and Triplet Global
Week 2: Alternate Panel
Week 3: Simple Triplet
Week 4: Triplet Complex

Follow-up calendar:
- Check with an X when the training is done and successfully completed.
- Otherwise enter the number of words correctly written in the box.
- Write 0 if the training has not been done.

	Day 1	Day 2	Day 3	Day 4	Day 5
Week 1					
Week 2					
Week 3					
Week 4					

At the end of the training (2 months), bring the completed card back to your speech therapist.

Remarks:

- Didi you child willingly complete the exercises: yes, no?
- What exercises did he or she like?
- What exercises did he or she dislike?
- What problems did you encounter and with what software?
- Other remarks:

First name:
Last name: Date:
