# Supplementary material for: Multimodal intervention in 8- to 13-year-old French dyslexic readers: Study protocol for a randomized multicenter controlled crossover trial
Source: BMC Pediatr. 2022 Dec 28;22:741. doi: 10.1186/s12887-022-03701-8 (PMC9795620; doi:10.1186/s12887-022-03701-8)
Supplement: Supplementary file 5 — Additional file 5. Parent sheet, cross-modal training (guidelines and protocol monitoring). [file 12887_2022_3701_MOESM5_ESM.pdf]

Parent sheet, cross-modal training

DDMR Study, 19-HPNCL-02

- Regularly remind your child why he or she needs to do the exercises.
- The speech therapist will tell you which exercises are appropriate for your child's age and difficulties. All you have to do is follow this progression.

Remember that regular practice guarantees effectiveness. So don't replace 2 days (15 min each) with 1 day (30 minutes).

Grapholearn and ARMAR: 15 min/day, 5 days/week

What for? When we identify sounds and letters at the same time, we read and write faster.

When? For 2 months, **5 days a week** for **15 minutes:**

- 10 minutes of Grapholearn training (level prescribed by the speech therapist).
- 5 minutes of ARMAR training.

How?

1. **Grapholearn (GL) training** is done on the computer. Start at the level prescribed by the speech therapist. Progress is determined by the program.
2. **ARMAR training** (accompanied, repeated, masked, and accelerated reading) training is done with an adult:

The child chooses a book that he or she wants to read or is reading and which he or she likes (no comics).
The child reads a text aloud <u>for 2 minutes.</u> The parent notes the number of errors in the table below (follow-up calendar).
The parent makes sure that the text is properly understood (by asking questions, responding to them if necessary and explaining unknown words).

5 minutes a day at home, 5 days/week

1) The parent reads the chosen text aloud at normal speed and with intonation while the child follows the written text with a pen, under the parent’s supervision (binary audio-visual processing).
2) The parent and the child read aloud at the same time (accompanied reading) and the child follows the text with a pen (binary audio-visual processing).
3) The child reads alone with a pen and the parent corrects the wrong words or reads words that are not read quickly enough.
4) The child reads aloud alone while listening to the music of his or her choice with a headset. This auditory masking increases reading fluidity by inhibiting the phonological pathway.
5) The child reads the text alone without masked hearing as quickly as possible. The parent notes the time and number of errors.

If the child wishes to continue reading the book silently, he or she can do so, but it is not an obligation.

The next day, the same exercise is repeated following the same protocol. The child reads the text for 2 minutes starting from where he or she stopped the day before. The parent notes the number of

errors, makes sure the text is properly understood and continues with the five steps described above.

Follow-up calendar:

- Write the number of errors at the end of each session in the corresponding box (GL - ARMAR)

	Day 1	Day 2	Day 3	Day 4	Day 5
Week 1					
Week 2					
Week 3					
Week 4					
	Day 1	Day 2	Day 3	Day 4	Day 5
Week 1					
Week 2					
Week 3					
Week 4					

At the end of the training (2 months), bring the completed card back to your speech therapist.

Comments:

- Did your child willingly complete the exercises: yes or no?
- What exercises did he or she like?
- What exercises did he or she dislike?
- What problems did you encounter and with what software?
- Other remarks:

First name:
Last name:
Date:
