# Supplementary material for: Multimodal intervention in 8- to 13-year-old French dyslexic readers: Study protocol for a randomized multicenter controlled crossover trial
Source: BMC Pediatr. 2022 Dec 28;22:741. doi: 10.1186/s12887-022-03701-8 (PMC9795620; doi:10.1186/s12887-022-03701-8)
Supplement: Supplementary file 6 — Additional file 6. Case Report Form Test 1 (Assessment and data entry). [file 12887_2022_3701_MOESM6_ESM.pdf]

Centre Hospitalier Universitaire de Nice
UNIVERSITÉ CÔTE D'AZUR

Case Report Form (CRF)

Phase 1- Test 1

Selection and Inclusion

Developmental dyslexia and method of remediation (DDMR) Multimodal intervention in French-speaking children aged 8 to 13 years: study protocol for a randomized multicentre controlled cross-over trial

DDMR
Internal Code: ID No. RCB 2019-A01453-54 – No. 19-HPNCL-02

Case Report Form (CRF)

Phase 1 - Test 1: Selection and Inclusion

Center no.: |__|__|

Patient no. ***(or patient code):*** **|__|__|**

Patient initials: |__| - |__|
(1st letter of last name – 1st letter of first name)

Sponsor:
Fondation LENVAL Hôpitaux Pédiatriques de Nice
CHU-Lenval
57, Avenue de la Californie - 06200 Nice
☏ : 04 92 03 03 92 🖷: 04 92 03 03 44
@ : fondation@lenval.com

Coordinating Investigator:
Karine Harrar Eskinazi
CERTA- Nice-CHU-Lenval
54 avenue de La Californie, 06200 Nice
☏ : 04 92 03 92 *00* 🖷: 04 92 03 *00 00*
@ : karine.eskinazi@univ-cotedazur.fr

Thesis director:
Pr Sylvane FAURE
Laboratoire LAPCOS, UCA – MSHS - SE
24, avenue des Diables Bleus, 06357 NICE Cedex
☏ : +33 4 89 15 23 72
@ : sylvane.faure@univ-cotedazur.fr

Thesis co-director:
Dr Bruno De Cara, MCF
Laboratoire LAPCOS, UCA – MSHS - SE
24, avenue des Diables Bleus, 06357 NICE Cedex
☏ : +33 4 89 15 23 72
@ : bruno.de-cara@univ-cotedazur.fr

INSTRUCTIONS TO COMPLETE THE CASE REPORT FORM

1-<u>GENERALITIES</u>:

- Write in the designated space.
- Write in capitals.
- Use a black ballpoint pen.
- Do not use abbreviations.
- Complete the header on each page.

2-<u>BOXES</u>:

Open boxes (|__|__|__|): enter only one character per box.
Closed boxes: check the corresponding answer ☒

- Numeric values:
  - Align the numerical values to the right
  - Do not leave boxes empty, enter zero. E.g.: Incorrect: |_2_|_1_|__| Correct: |_0_|_2_|_1_|
- Dates: write dates in the day/month/year format (dd/mm/yyyy).

3-<u>ERRORS</u>:

In the event of an error, cross out the erroneous information with a single line so that it remains visible. Note the correction next to it, report, and date. Do not use correction fluid.

4-<u>END OF STUDY</u>:

When the patient's participation ends, whether at the end of the study or prematurely, complete the end-of-study sheet.

- **The end-of-study date:**

It is the date of the last information collected as part of the study. No review related to the study, administration of the treatment under study, or data collection will be carried out after that date. The end-of-study date may be the date of the last visit or the date of the last information collected as part of the visit.

- **Early exit:**

If the patient leaves the study before the end of the study, please specify the reason for this premature exit. If in doubt do not hesitate to contact the sponsor CRA (Clinical Research Associate) or the coordinating investigator. Premature exit by the patient: the patient may decide at any time to interrupt his or her participation in the study (refusal to continue / withdrawal of consent). All the data collected until the exit will be collected and analyzed (Jardé Law – Article L.1121-1-1du CSP, 18th November 2016 version).

5-<u>CONTACT</u>:

If any problems arise when filling out the CRF, please contact the sponsor (contact details mentioned on page 1).

6-<u>Specific conventions for filling out the CRF</u>:

- The data is collected in the tables included in this CRF in raw scores, Z scores and percentiles according to the instructions provided in the manuals for each test.
- For children with "high intellectual potential" the reading age must be compared with the age of development and not with the actual age.
- For Alouette©, two calibrations are used: the first calibration for the reading age and the second calibration dating from 2005.
- For DeltaText and Likert scales, there are no Z scores.
- Training is adapted to the cognitive profile of each child. For example, the child that does not have a visuo-attentional span disorder will complete 2 months of training with Switchipido© and will not practice with Maeva©. Therefore, the pre- and post-training baseline tables for Maeva© will not be used.

Experimental procedure

PHASES		Visits	Details and duration of the assement or intervention	
Phase 1	**TEST 1**	V 1 to V4	Information, consent, selection<br>**Baseline assessment**	4 hr
	**No intensive training 2 months**	V 5 to V 12	Speech therapy sessions (STS): 30 min/week	STS: 4 hr
	**TEST 2**	V 13- V 14	**Post-control assessment**	2 hr
Phase 2	**INTENSIVE TRAINING 1 2 months**	V 15	Pre-training 1A baseline assessment	30 min
		V 16 to V 19	Home training (HT): 5 days/week-15 min/day<br>Speech therapy sessions (STS): 30 min/week	HT: 5 hr<br>STS: 2 hr
		V 20	Post-training 1A baseline assessment	15 min
		V 21	Pre-training 1B baseline assessment	30 min
		V 22 to V 25	Home training (HT): 5 days/week-15 min/day<br>Speech therapy sessions (STS): 30 min/week	HT: 5 hr<br>STS: 2 hr
		V 26	Post-training 1B baseline assessment	10 min
	**TEST 3**		**Post-training assessment 1**	50 min
	**Break**	V 27, V 28	15 days without home training	1 hr
Phase 2	**INTENSIVE TRAINING 2 2 months**	V 29	Pre-training 2A baseline assessment	30 min
		V 30 to V 33	Home training (HT): 5 days/week-15 min/day<br>Speech therapy sessions (STS): 30 min/week	HT: 5 hr<br>STS: 2 hr
		V 34	Post-training 2A baseline assessment	15 min
		V 35	Pre-training 2B baseline assessment	30 min
		V 36 to V 39	Home training (HT): 5 days/week-15 min/day<br>Speech therapy sessions (STS): 30 min/week	HT: 5 hr<br>STS: 2 hr
		V 40	Post-training 2B baseline assessment	10 min
	**TEST 4**	V 40	**Post-training assessment 2**	50 min
	**Break**	V 41, V 42	15 days without home training	1 hr
Phase 2	**INTENSIVE TRAINING 3 2 months**	V 43	Pre-training 3 baseline assessment	30 min
		V 44 to V 51	Home training (HT): 5 days/week-15 min/day<br>Speech therapy sessions (STS): 30 min/week	HT: 10 hr<br>STS: 4 hr
		V 52	Post-training 3 baseline assessment	10 min
	**TEST 5**	V 53-54	**Post-training assessment 3**	2 hr
Phase 3	**No intensive training 2 months**	V 56 to V 64	Stop home training<br>Speech therapy sessions (STS): 30 min/week	STS: 4 hr
	**TEST 6**	V 65	**Follow-up assessment**	15 min
Total		65 weeks	16 months	49 hr 20 min

SELECTION VISIT- PHASE 1-Test 1-V 1

V1: first visit: 1 hour

Date : |__|__| / |__|__| / |__|__|__|__| (DD/MM/YYYY)

CONSENT COLLECTION

❑ Mother and ❑ Father

on |__|__| / |__|__| / |__|__|__|__| on |__|__| / |__|__| / |__|__|__|__|
DD/MM/YYYY ***DD/MM/YYYY***

Or

❑ The holder of parental authority on |__|__| / |__|__| / |__|__|__|__| (***DD/MM/YYYY)***

Note in the patient's source file the patient's participation in the study and the patient's consent.

In the absence of one of the parents, a prior oral agreement of the absent parent must be obtained and notified in the patient's medical file.

Contact information and written consent of the absent parent should be collected as soon as possible.

If one of the parents has not signed the consent form, the investigator must provide evidence in the child's source file of having tried to contact the parent.

Investigator's name: ____

Signature:

DEMOGRAPHICS

Born on: |__|__|/|__|__|/|__|__|__|__| (***DD/MM/YYYY)*** Age: |__|__| years |__|__| months

Sex: ❑ Male ❑ Female Grade: |__|__|__|

HISTORY

- Reason for speech therapy: Reading difficulties ❑ Spelling difficulties ❑

  School difficulties❑

  Other..

- Illnesses: ❑Yes ❑ No If yes which ones? (allergies, frequent ear infections, cranial trauma, seizures, neurological or genetic disorders, other)
  ....
  ....

- Drug treatment: ❑Yes No ❑ If yes, which ones? (treatment for behavioral disorders or hyperactivity) ....
- Medical history: ❑Yes ❑ No If yes, describe (allergies, frequent ear infections, cranial trauma, epileptic seizures, neurological or genetic disorders, other)
  ....
  ....
- Eye examination: ❑Yes ❑ No Hearing test: ❑Yes ❑ No
- Laterality for writing: Right hand ❑ Left hand ❑
- Native Language: French ❑
  Other..
- Mother's language .... Father's language..........................
- Family history:

	Age	Types of difficulties	Degrees	Profession
Mother				
Father				
Sibling				
Sibling				
Sibling				
Other				

SELECTION VISIT- PHASE 1 - V 1

ASSOCIATED DISORDERS

Has the patient undergone or is the patient currently undergoing any other treatment or assessment (speech therapy, neuropsychology, clinical psychology, orthoptia, occupational therapy, psychomotricity, other)

❑ Yes ❑ No

➯ *If so, please complete the sheet of concurrent treatments at the end of the CRF*

DEVELOPMENT

Pathological pregnancy ❑Yes ❑ No

If so, why? (medication, medical problems, infectious episodes, toxic products, other)

....

Pathological birth ❑Yes ❑ No

If so, why? (term, caesarean section, forceps, apgar, intensive care, other)

………

Disorders during childhood ❑Yes ❑ No

If so, which ones (sleep disturbance, eating, cleanliness, incessant crying, other)

………

- Sitting age:
- Walking age:
- Age of first words:
- Age of first sentences:

Impaired global motor skills ❑Yes ❑ No

If so, which ones (running, jumping, cycling, other)

………

Impaired fine motor skills ❑Yes ❑ No

If so, which ones (tie laces, fasten buttons, cut, embed, use cutlery, write, other)

………

Impaired building skills ❑ Yes ❑No

If so, which ones (puzzles, LEGO©, models, other)

………

Language skill disorders ❑Yes ❑ No

If so, which ones (vocabulary, sentences, narrative, other)

………

Oral comprehension disorder ❑Yes ❑ No

If so, in what context (simple language, elaborate language, school, home, multiple instructions)

………

Impaired attentional abilities ❑Yes ❑ No

If so, in what context (sustained activity, film, game, school, homework, other)

………

SCHOOL SITUATION

- In kindergarten, difficulties in carrying out activities ❑Yes ❑ No
- Difficulties in learning to read ❑Yes ❑ No
- Difficulties in learning to spell ❑Yes ❑ No
- Difficulties in learning to write ❑Yes ❑ No
- Difficulties in learning mathematics ❑Yes ❑ No
- Current persistence of reading difficulties ❑Yes ❑ No
- Difficulties in understanding written instructions ❑Yes ❑ No
- Current persistence of spelling difficulties ❑Yes ❑ No
- Current persistence of difficulties in mathematics ❑Yes ❑ No

- Recommendation to repeat the school year ❑Yes ❑ No
- Repeated school year, which one ....❑Yes ❑ No
- Gifted student, in which areas...❑Yes ❑ No
- Remedial assistance ❑Yes ❑ No
- Help with homework ❑Yes ❑ No
- Time devoted to homework in the evening

....

- Additional comments

....
....

SELECTION TESTS, READING AND SPELLING

For the administration of the tests, refer to the instruction manual

- Reading meaningless text (Alouette©)
- Spelling (Chronosdictées©, version A)

SELECTION CRITERIA

SELECTION CRITERIA	Yes	No
Age ≥ 8 and ≤ 13	❑	❑
Diagnosis of dyslexia or difficulty in reading and/or spelling	❑	❑
Absence of perceptual, neurological or behavioral disorders	❑	❑
No oral language problems	❑	❑
Native language: French or early school in French	❑	❑
No attentional problems	❑	❑
No rehabilitation is underway	❑	❑
Home equipped with a computer connected to internet for daily training	❑	❑
Informed consent signed by both parents or the representative of the parental authority	❑	❑
Social Security affiliation or beneficiary	❑	❑

If one or more "NO" boxes are checked the patient is not selected for the study

DATA COLLECTION-V 1

<table>
<tr><th rowspan="19">PHASE 1

TEST 1

V 1</th><th colspan="2">Tests</th><th>Data</th><th>Raw scores</th><th>Rate + or -</th><th>Z scores (SD or percentiles)</th><th>Time (min)</th></tr>
<tr><td>Anamnesia</td><td>Survey (CRF p.5 to 7)</td><td colspan="4">"Don't enter anything here"</td><td>15</td></tr>
<tr><td rowspan="7">Reading meaningless text</td><td rowspan="7">Alouette©</td><td>Number of errors</td><td>|__|__|</td><td></td><td>|__|__|.|__|__|</td><td rowspan="7">3</td></tr>
<tr><td>Number of words read</td><td>|__|__|__|</td><td></td><td>|__|__|.|__|__|</td></tr>
<tr><td>Number of correct words</td><td>|__|__|__|</td><td></td><td>|__|__|.|__|__|</td></tr>
<tr><td>Time in seconds</td><td>|__|__|__|</td><td></td><td>|__|__|.|__|__|</td></tr>
<tr><td>Accuracy index</td><td>|__|__|__|</td><td></td><td>|__|__|.|__|__|</td></tr>
<tr><td>Speed index</td><td>|__|__|__|</td><td></td><td>|__|__|.|__|__|</td></tr>
<tr><td>Reading age</td><td colspan="3">|__|__| years |__|__| months</td></tr>
<tr><td rowspan="6">Spelling test</td><td rowspan="6">Chronosdictées©
Version A</td><td>Number of phonetic errors</td><td>|__|__|</td><td></td><td>|__|__|.|__|__|</td><td rowspan="6">10</td></tr>
<tr><td>Number of lexical errors</td><td>|__|__|</td><td></td><td>|__|__|.|__|__|</td></tr>
<tr><td>Number of grammatical errors</td><td>|__|__|</td><td></td><td>|__|__|.|__|__|</td></tr>
<tr><td>Number of omissions</td><td>|__|__|</td><td></td><td>|__|__|.|__|__|</td></tr>
<tr><td>Number of segmentation errors</td><td>|__|__|</td><td></td><td>|__|__|.|__|__|</td></tr>
<tr><td>Total number of errors</td><td>|__|__|</td><td></td><td>|__|__|.|__|__|</td></tr>
<tr><td>Selection</td><td>Table CRF p. 8</td><td colspan="4">"Don't enter anything here"</td><td>5</td></tr>
<tr><td>Information</td><td>CRF</td><td colspan="4">"Don't enter anything here"</td><td>20</td></tr>
<tr><td>Consent</td><td>CRF</td><td colspan="4">"Don't enter anything here"</td><td>5</td></tr>
<tr><td colspan="6">Total time (min)</td><td>58</td></tr>
</table>

INCLUSION VISIT - PHASE 1- TEST 1- V 2 to V 4

To administer and determine the order of tests, refer to the manual sent to you that specifies the order to be followed.

INCLUSION VISIT V 2

V2: second visit: 1 hour

Date : |__|__| / |__|__| / |__|__|__|__| ***(DD/MM/YYYY)***

- Reading aloud a meaningful text, La Mouette (Evaléo© 6-15)
- Phonological analysis, non-word repetition and rapid automatized naming (Evalec©)
- Visuo-attentional span (Evadys©)
- Oral comprehension (ECOSSE©) - only if oral language has not been tested or if there is still any doubt about a possible "specific language impairment"

<table>
<tr><th>Internal code</th><th>Patient number</th><th rowspan="2">PHASE 1 - Selection and inclusion
TEST 1</th></tr>
<tr><td>19-HPNCL-02</td><td>|__|__| / |__|__| / |__|__|__|__|</td></tr>
</table>

<table>
<tr><td rowspan="23">Phase 1

TEST 1

V 2</td><th colspan="2">Tests</th><th>Data</th><th>Raw scores</th><th>Rate + or -</th><th>Z scores (SD or percentiles)</th><th>Time min</th></tr>
<tr><td rowspan="4">Reading meaningful text EVALEO©</td><td rowspan="4">"Mouette"</td><td>Number of words read</td><td>|__|__|__|</td><td></td><td>|__|</td><td rowspan="4">2</td></tr>
<tr><td>Number of correct words</td><td>|__|__|__|</td><td></td><td>|__|</td></tr>
<tr><td>% correct/read</td><td>|__|__|__|.|__|</td><td></td><td>|__|</td></tr>
<tr><td>Degradation index</td><td>|__|__|__|</td><td></td><td>|__|</td></tr>
<tr><td rowspan="12">Phonological awarness EVALEC©</td><td rowspan="2">Syllabe suppression</td><td>Accuracy (% errors)</td><td>|__|__|__|__|.|__|__|</td><td></td><td>|__|__|.|__|__|</td><td rowspan="6">8</td></tr>
<tr><td>Time in seconds</td><td>|__|__|__|__|.|__|__|</td><td></td><td>|__|__|.|__|__|</td></tr>
<tr><td rowspan="2">Phoneme suppression CVC</td><td>Accuracy (% errors)</td><td>|__|__|__|__|.|__|__|</td><td></td><td>|__|__|.|__|__|</td></tr>
<tr><td>Time in seconds</td><td>|__|__|__|__|.|__|__|</td><td></td><td>|__|__|.|__|__|</td></tr>
<tr><td rowspan="2">Phoneme suppression CCV</td><td>Accuracy (% errors)</td><td>|__|__|__|__|.|__|__|</td><td></td><td>|__|__|.|__|__|</td></tr>
<tr><td>Time in seconds</td><td>|__|__|__|__|.|__|__|</td><td></td><td>|__|__|.|__|__|</td></tr>
<tr><td rowspan="2">Non-word repetition</td><td>Span</td><td>|__|</td><td></td><td>|__|__|.|__|__|</td><td rowspan="2">5</td></tr>
<tr><td>Time in seconds</td><td>|__|__|__|__|.|__|__|</td><td></td><td>|__|__|.|__|__|</td></tr>
<tr><td rowspan="4">Rapid automatized naming</td><td>Color recognition, %</td><td>|__|__|__|__|.|__|__|</td><td></td><td>|__|__|.|__|__|</td><td rowspan="2">2</td></tr>
<tr><td>Color recognition, time in seconds</td><td>|__|__|__|__|.|__|__|</td><td></td><td>|__|__|.|__|__|</td></tr>
<tr><td>Reading names, %</td><td>|__|__|__|__|.|__|__|</td><td></td><td>|__|__|.|__|__|</td><td></td></tr>
<tr><td>Reading names, time in seconds</td><td>|__|__|__|__|.|__|__|</td><td></td><td>|__|__|.|__|__|</td><td></td></tr>
<tr><td rowspan="5">Visuo-attentional span EVADYS©</td><td>Letter threshold</td><td>Threshold in ms</td><td>|__|__|__|</td><td></td><td>|__|__|.|__|__|</td><td rowspan="5">20</td></tr>
<tr><td rowspan="2">Global Report GR</td><td>GR Score</td><td>|__|__|__|</td><td></td><td>|__|__|.|__|__|</td></tr>
<tr><td>GR Span</td><td>|__|.|__|__|</td><td></td><td>|__|__|.|__|__|</td></tr>
<tr><td rowspan="2">Partial Report PR</td><td>PR Score</td><td>|__|__|</td><td></td><td>|__|__|.|__|__|</td></tr>
<tr><td>PR Span</td><td>|__|.|__|__|</td><td></td><td>|__|__|.|__|__|</td></tr>
<tr><td>Oral comprehension</td><td>ECOSSE©</td><td>Number of errors</td><td>|__|__|</td><td></td><td>|__|__|</td><td>15</td></tr>
<tr><td colspan="6">Total time (min)</td><td>52</td></tr>
</table>

INCLUSION VISIT V 3

V3: third visit: 1 hour

Date: |__|__| / |__|__| / |__|__|__|__| ***(DD/MM/YYYY)***

- Local/global analysis (SIGL©)
- Reading aloud regular, irregular words, and non-words (Evalec©)
- Matrix Reasoning (Wisc V©) - only if the neuropsychological check-up has not been performed or if it is not planned
- Perception of the evolution of the disorder, 2 questionnaires for parents and children (Likert scale)

<table>
<tr><th>Internal code</th><th>Patient number</th><th rowspan="2">PHASE 1 - Selection and inclusion
TEST 1</th></tr>
<tr><td>19-HPNCL-02</td><td>|__|__| / |__|__| / |__|__|__|__|</td></tr>
</table>

<table>
<tr><th rowspan="26">Phase 1

TEST 1

V3</th><th colspan="2">Tests</th><th>Data</th><th>Raw Scores</th><th>Rate + or -</th><th>Z scores (SD or percentiles)</th><th>Time min</th></tr>
<tr><td rowspan="6">Global/local analysis SIGL© "Drawings"</td><td rowspan="3">Response time (RT)</td><td>Local RT (ms)</td><td>|__|__|__|.|__|__|</td><td></td><td>|__|__|.|__|__|</td><td rowspan="6">20</td></tr>
<tr><td>Global RT (ms)</td><td>|__|__|__|.|__|__|</td><td></td><td>|__|__|.|__|__|</td></tr>
<tr><td>Interference asymmetry RT (ms)</td><td>|__|__|__|.|__|__|</td><td></td><td>|__|__|.|__|__|</td></tr>
<tr><td rowspan="3">Error Rate (ER)</td><td>Local ER (%)</td><td>|__|__|__|.|__|__|</td><td></td><td>|__|__|.|__|__|</td></tr>
<tr><td>Global ER (%)</td><td>|__|__|__|.|__|__|</td><td></td><td>|__|__|.|__|__|</td></tr>
<tr><td>Interference asymmetry ER (%)</td><td>|__|__|__|.|__|__|</td><td></td><td>|__|__|.|__|__|</td></tr>
<tr><td rowspan="14">Word reading EVALEC©</td><td rowspan="2">Regular words (RW)</td><td>Accuracy (% errors)</td><td>|__|__|__|.|__|__|</td><td></td><td>|__|__|.|__|__|</td><td rowspan="14">15</td></tr>
<tr><td>Time in ms</td><td>|__|__|__|__|</td><td></td><td>|__|__|.|__|__|</td></tr>
<tr><td rowspan="2">Irregular words (IW)</td><td>Accuracy (% errors)</td><td>|__|__|__|.|__|__|</td><td></td><td>|__|__|.|__|__|</td></tr>
<tr><td>Time in ms</td><td>|__|__|__|__|</td><td></td><td>|__|__|.|__|__|</td></tr>
<tr><td rowspan="2">Non-words (NW)</td><td>Accuracy (% errors)</td><td>|__|__|__|.|__|__|</td><td></td><td>|__|__|.|__|__|</td></tr>
<tr><td>Time in ms</td><td>|__|__|__|__|</td><td></td><td>|__|__|.|__|__|</td></tr>
<tr><td rowspan="2">Short IW</td><td>Accuracy (% errors)</td><td>|__|__|__|.|__|__|</td><td></td><td>|__|__|.|__|__|</td></tr>
<tr><td>Time in ms</td><td>|__|__|__|__|</td><td></td><td>|__|__|.|__|__|</td></tr>
<tr><td rowspan="2">Long IW</td><td>Accuracy (% errors)</td><td>|__|__|__|.|__|__|</td><td></td><td>|__|__|.|__|__|</td></tr>
<tr><td>Time in ms</td><td>|__|__|__|__|</td><td></td><td>|__|__|.|__|__|</td></tr>
<tr><td rowspan="2">Short NW</td><td>Accuracy (% errors)</td><td>|__|__|__|.|__|__|</td><td></td><td>|__|__|.|__|__|</td></tr>
<tr><td>Time in ms</td><td>|__|__|__|__|</td><td></td><td>|__|__|.|__|__|</td></tr>
<tr><td rowspan="2">Long NW</td><td>Accuracy (% errors)</td><td>|__|__|__|.|__|__|</td><td></td><td>|__|__|.|__|__|</td></tr>
<tr><td>Time in ms</td><td>|__|__|__|__|</td><td></td><td>|__|__|.|__|__|</td></tr>
<tr><td>Wisc 5©</td><td>Matrix Reasoning</td><td>Correct items /32</td><td>|__|__|</td><td></td><td>|__|__|</td><td>10</td></tr>
<tr><td rowspan="2">Surveys-Likert scale</td><td>Child pre-test</td><td>Total points</td><td>|__|__|</td><td></td><td></td><td rowspan="2">5</td></tr>
<tr><td>Parents pre-test</td><td>Total points</td><td>|__|__|</td><td></td><td></td></tr>
<tr><td colspan="6">Total time (min)</td><td>55</td></tr>
</table>

V4 INCLUSION VISIT

V4: fourth visit: 1 hour

Date : |__|__| / |__|__| / |__|__|__|__| ***(DD/MM/YYYY)***

- Short-term verbal memory and working memory, digit span (Evaléo©)
- Vocabulary (Evaléo©) - only if oral language has not been tested
- Visuo-spatial span (Corsi©)
- Reading comprehension (Orlec© L3)
- 2 minutes reading words, Eval2M (Evaléo©)
- Reading aloud a meaningless text (Deltatext© 1)

<table>
<tr><th>Internal code</th><th>Patient number</th><th rowspan="2">PHASE 1 - Selection and inclusion
TEST 1</th></tr>
<tr><td>19-HPNCL-02</td><td>|__|__| / |__|__| / |__|__|__|__|</td></tr>
</table>

<table>
<tr><th rowspan="21">PHASE 1

TEST 1

V4</th><th colspan="2">Tests</th><th>Data</th><th>Raw Scores</th><th>Rate + or -</th><th>Z scores (SD or percentiles)</th><th>Time min</th></tr>
<tr><td rowspan="4">Digit span</td><td rowspan="4">EVALEO©</td><td>Score forward span</td><td>|__|</td><td></td><td>|__|</td><td rowspan="4">8</td></tr>
<tr><td>Forward span</td><td>|__|</td><td></td><td>|__|</td></tr>
<tr><td>Score backward span</td><td>|__|</td><td></td><td>|__|</td></tr>
<tr><td>Backward span</td><td>|__|</td><td></td><td>|__|</td></tr>
<tr><td rowspan="3">Vocabulary EVALEO©</td><td rowspan="2">Picture Naming</td><td>Accuracy (correct iERms)</td><td>|__|__|</td><td></td><td>|__|</td><td rowspan="3">15</td></tr>
<tr><td>Total time, in seconds</td><td>|__|__|__|.|__|</td><td></td><td>|__|</td></tr>
<tr><td>Picture Designation</td><td>Accuracy (correct iERms)</td><td>|__|__|__|</td><td></td><td>|__|</td></tr>
<tr><td rowspan="2">Visuo-spatial span</td><td rowspan="2">Corsi©</td><td>Forward span</td><td>|__|</td><td></td><td>|__|__|.|__|__|</td><td rowspan="2">8</td></tr>
<tr><td>Backward span</td><td>|__|</td><td></td><td>|__|__|.|__|__|</td></tr>
<tr><td rowspan="2">WritERn comprehension</td><td rowspan="2">Orlec, L3©</td><td>Accuracy (correct items)</td><td>|__|__|</td><td></td><td>|__|__|.|__|__|</td><td rowspan="2">5</td></tr>
<tr><td>Correct items (%)</td><td>|__|__|__|.|__|__|</td><td></td><td>|__|__|.|__|__|</td></tr>
<tr><td rowspan="3">Reading meaningless text</td><td rowspan="3">Deltatext© 1</td><td>Number of words read</td><td>|__|__|__|</td><td></td><td></td><td rowspan="3">3</td></tr>
<tr><td>Number of errors</td><td>|__|__|__|</td><td></td><td></td></tr>
<tr><td>Time in seconds</td><td>|__|__|__|</td><td></td><td></td></tr>
<tr><td rowspan="3">Words readi in 2 min</td><td rowspan="3">Eval 2M©</td><td>Number of words read</td><td>|__|__|__|</td><td></td><td>|__|</td><td rowspan="3">2</td></tr>
<tr><td>Number of correct words</td><td>|__|__|__|</td><td></td><td>|__|</td></tr>
<tr><td>% correct/read</td><td>|__|__|__|.|__|__|</td><td></td><td>|__|</td></tr>
<tr><td colspan="6">Total time (min)</td><td>40</td></tr>
</table>

INCLUSION AND NON-INCLUSION CRITERIA

INCLUSION CRITERIA	Yes	No
Age greater than or equal to 8 years and less than or equal to 13 years.	❑	❑
Diagnosis of dyslexia validated by performance lower than 1.5 standard deviation from the average developmental age on the Alouette© test and the reading tasks of irregular words and non-words (Evalec©)	❑	❑
Performance lower or equal to -1.5 standard deviation from the average in at least one phonological task (Evalec©) and at least one visuo-attentional task (Sigl©, Evadys©)	❑	❑
Home equipped with a computer connected to internet for daily training	❑	❑
Informed consent signed by one of the parents or the representative of the parental authority	❑	❑
Social Security affiliate or beneficiary	❑	❑

If one or more "NO" boxes are checked the patient is not included in the study

NON-INCLUSION CRITERIA	Yes	No
Intellectual delay or neurological disorders or global developmental disorder	❑	❑
Primary sensory deficit	❑	❑
Educational deficiencies	❑	❑
Attention Deficit Disorder with or without Hyperactivity (ADHD)	❑	❑
Specific oral language impairment	❑	❑
Previous daily phonological or visuo-attentional training	❑	❑

If one or more "YES" boxes are checked the patient is not included in the study

Is the patient included in the study? Yes ❑ No ❑

If not, why: ....

Comments: ....

RANDOMIZATION

At the end of the inclusion visits, send your randomization sheet to: karine.eskinazi@wanadoo.fr

The coordinating investigator will tell you whether participants are assigned to group 1 or group 2 and will send you the appropriate CRFs following the study.

- Group 1: PHONO 1 - VA 2- IM 3
- Group 2: VA 1- PHONO 2- IM 3

The participant is not informed of his or her attribution to group 1 or 2 and will follow the instructions given by the associate investigative speech therapist regarding the conduct of the study.

Rating: Treatment group: 1❑ 2❑

Randomization No.:

Center No. :

Internal code	Patient number	PHASE 1 - Selection and inclusion TEST 1
19-HPNCL-02	|__|__| / |__|__| / |__|__|__|__|	

	Treatments				DIAGNOSIS	Start (DD/MM/YYYY)	End Note "Ongoing" if the treatment is not finished at the end of the study	
	TYPES	CHECK-UP DATES (DD/MM/YYYY)	Rehabilitation Yes	Rehabilitation No			End Date (JJ/MM/AAAA)	Ongoing
1	………………	|__|__| / |__|__| / |__|__|__|__|	❑	❑	……………………………………	|__|__| / |__|__| / |__|__|__|__|	|__|__| / |__|__| / |__|__|__|__|	❑
2	………………	|__|__| / |__|__| / |__|__|__|__|	❑	❑	……………………………………	|__|__| / |__|__| / |__|__|__|__|	|__|__| / |__|__| / |__|__|__|__|	❑
3	………………	|__|__| / |__|__| / |__|__|__|__|	❑	❑	……………………………………	|__|__| / |__|__| / |__|__|__|__|	|__|__| / |__|__| / |__|__|__|__|	❑
4	………………	|__|__| / |__|__| / |__|__|__|__|	❑	❑	……………………………………	|__|__| / |__|__| / |__|__|__|__|	|__|__| / |__|__| / |__|__|__|__|	❑
5	………………	|__|__| / |__|__| / |__|__|__|__|	❑	❑	……………………………………	|__|__| / |__|__| / |__|__|__|__|	|__|__| / |__|__| / |__|__|__|__|	❑
6	………………	|__|__| / |__|__| / |__|__|__|__|	❑	❑	……………………………………	|__|__| / |__|__| / |__|__|__|__|	|__|__| / |__|__| / |__|__|__|__|	❑

Thank you for postponing all treatment received except treatment provided in the study
