# Supplementary material for: Multimodal intervention in 8- to 13-year-old French dyslexic readers: Study protocol for a randomized multicenter controlled crossover trial
Source: BMC Pediatr. 2022 Dec 28;22:741. doi: 10.1186/s12887-022-03701-8 (PMC9795620; doi:10.1186/s12887-022-03701-8)
Supplement: Supplementary file 7 — Additional file 7. Case Report Form Group 1 (Assessment and data entry). [file 12887_2022_3701_MOESM7_ESM.pdf]

Centre Hospitalier Universitaire de Nice
UNIVERSITÉ CÔTE D'AZUR

Case Report Form (CRF)

Group 1: PHONO 1 / VA 2 / C M 3 training

Coordinating center

Delegation for Clinical Research and Innovation

Developmental dyslexia and methods of remediation (DDMR). Multimodal intervention in French-speaking children aged 8 to 13 years: study protocol for a randomized multicenter controlled crossover trial

DDMR
Internal Code: ID No. RCB 2019-A01453-54 – No. 19-HPNCL-02

Case Report Form (CRF)

GROUP 1: PHONO 1 / VA 2 / IM 3 training

Center no.: |__|__|

Patient no. ***(or patient code):*** **|__|__|**

Patient initials: |__| - |__|
(1st letter of last name – 1st letter of first name)

Sponsor:

Fondation LENVAL Hôpitaux Pédiatriques de Nice
CHU-Lenval
57, Avenue de la Californie – 06200 Nice
☏ : 04 92 03 03 92 🖷: 04 92 03 03 44
@ : fondation@lenval.com

Coordinating Investigator:

Karine Harrar Eskinazi
CERTA- Nice-CHU-Lenval
54 avenue de La Californie, 06200 Nice
☏ : 04 92 03 92 *00* 🖷: 04 92 03 *00 00*
@ : karine.eskinazi@univ-cotedazur.fr

Thesis director:

Pr Sylvane FAURE
Laboratoire LAPCOS, UCA – MSHS – SE
24, avenue des Diables Bleus, 06357 NICE Cedex
☏ : +33 4 89 15 23 72
@ : sylvane.faure@univ-cotedazur.fr

Thesis co-director:

Dr Bruno De Cara, MCF
Laboratoire LAPCOS, UCA – MSHS – SE
24, avenue des Diables Bleus, 06357 NICE Cedex
☏ : +33 4 89 15 23 72
@ : bruno.de-cara@univ-cotedazur.fr

INSTRUCTIONS TO COMPLETE THE CASE REPORT FORM

1-GENERALITIES:

- Write in the designated space.
- Write in capitals.
- Use a black ballpoint pen.
- Do not use abbreviations.
- Complete the header on each page.

2-BOXES:

Open boxes (|__|__|__|): enter only one character per box.
Closed boxes: check the corresponding answer ☒

- Numeric values:
  - Align the numerical values to the right
  - Do not leave boxes empty, enter zero. E.g.: Incorrect: |_2_|_1_|__| Correct: |_0_|_2_|_1_|
- Dates: write dates in the day/month/year format (dd/mm/yyyy).

3-ERRORS:

In the event of an error, cross out the erroneous information with a single line so that it remains visible. Note the correction next to it, report, and date. Do not use correction fluid.

4-END OF STUDY:

When the patient's participation ends, whether at the end of the study or prematurely, complete the end-of-study sheet.

- **The end-of-study date:**

It is the date of the last information collected as part of the study. No review related to the study, administration of the treatment under study, or data collection will be carried out after that date. The end-of-study date may be the date of the last visit or the date of the last information collected as part of the visit.

- **Early exit:**

If the patient leaves the study before the end of the study, please specify the reason for this premature exit. If in doubt do not hesitate to contact the sponsor CRA (Clinical Research Associate) or the coordinating investigator.
Premature exit by the patient: the patient may decide at any time to interrupt his or her participation in the study (refusal to continue / withdrawal of consent). All the data collected until the exit will be collected and analyzed (Jardé Law – Article L.1121-1-of the CSP, November 18, 2016 version).

5-CONTACT:

If any problems arise when filling out the CRF, please contact the sponsor (contact details mentioned on page 1).

6-Specific conventions for filling out the CRF:

- The data is collected in the tables included in this CRF in raw scores, Z scores and percentiles according to the instructions provided in the manuals for each test.
- For children with "high intellectual potential" the reading age must be compared with the age of development and not with the actual age.
- For Alouette©, two calibrations are used: the first calibration for the reading age and the second calibration dating from 2005.
- For DeltaText and Likert scales, there are no Z scores.
- Training is adapted to the cognitive profile of each child. For example, the child that does not have a visuo-attentional span disorder will complete 2 months of training with Switchipido© and will not practice with Maeva©. Therefore, the pre- and post-training baseline tables for Maeva© will not be used.

Timeline and experimental procedure

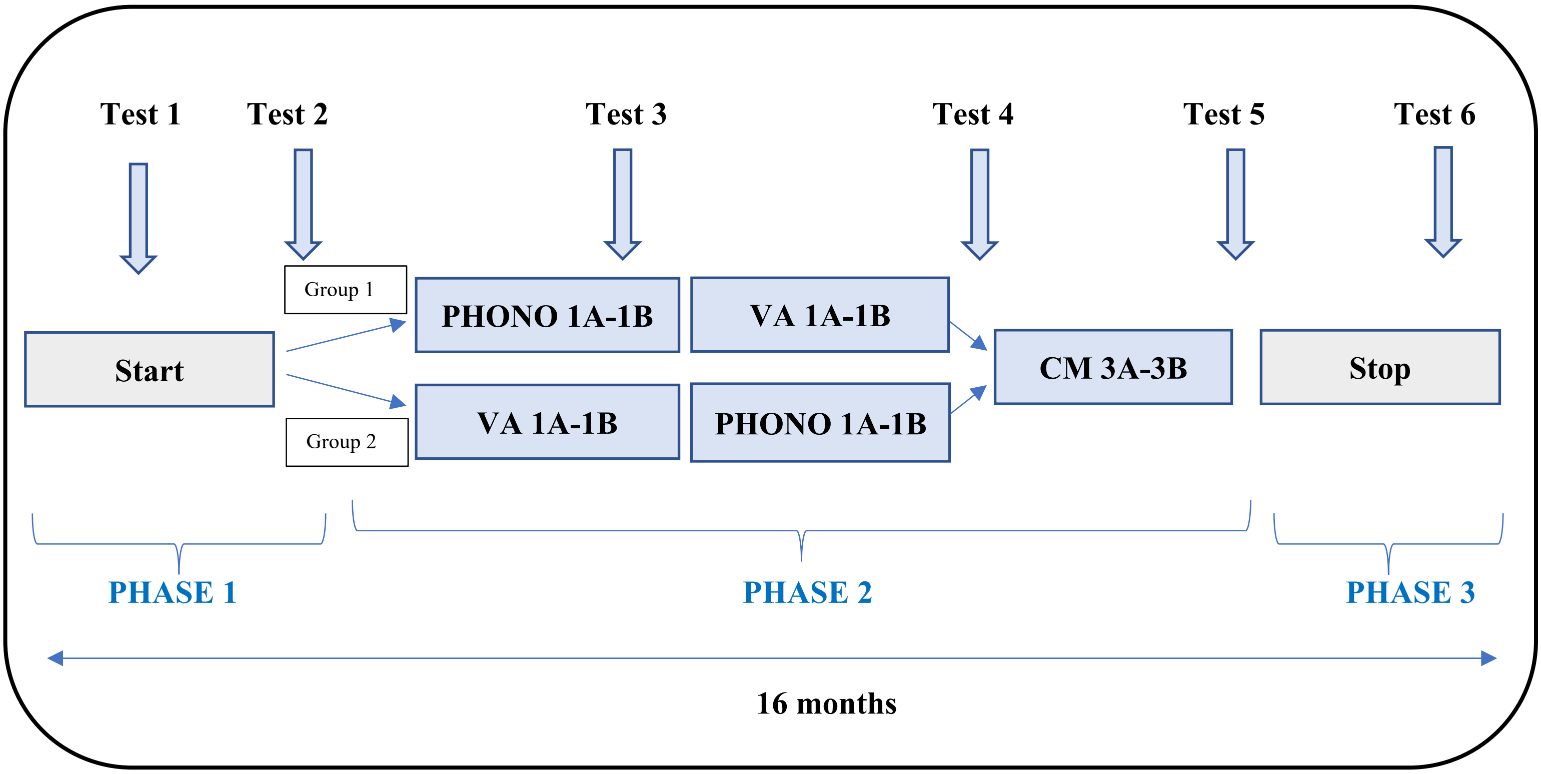

PHONO: phonological; VA: visuo-attentional; CM: cross-modal

PHASES		Visits	Details and duration of the assement or intervention	
Phase 1	**TEST 1**	V 1 to V4	Information, consent, selection<br>**Baseline assessment**	4 hr
	**No intensive training 2 months**	V 5 to V 12	Speech therapy sessions (STS): 30 min/week	STS: 4 hr
	**TEST 2**	V 13- V 14	**Post-control assessment**	2 hr
Phase 2	**INTENSIVE TRAINING 1 2 months**	V 15	Pre-training 1A baseline assessment	30 min
		V 16 to V 19	Home training (HT): 5 days/week-15 min/day<br>Speech therapy sessions (STS): 30 min/week	HT: 5 hr<br>STS: 2 hr
		V 20	Post-training 1A baseline assessment	15 min
		V 21	Pre-training 1B baseline assessment	30 min
		V 22 to V 25	Home training (HT): 5 days/week-15 min/day<br>Speech therapy sessions (STS): 30 min/week	HT: 5 hr<br>STS: 2 hr
		V 26	Post-training 1B baseline assessment	10 min
	**TEST 3**		**Post-training assessment 1**	50 min
	**Break**	V 27, V 28	15 days without home training	1 hr
Phase 2	**INTENSIVE TRAINING 2 2 months**	V 29	Pre-training 2A baseline assessment	30 min
		V 30 to V 33	Home training (HT): 5 days/week-15 min/day<br>Speech therapy sessions (STS): 30 min/week	HT: 5 hr<br>STS: 2 hr
		V 34	Post-training 2A baseline assessment	15 min
		V 35	Pre-training 2B baseline assessment	30 min
		V 36 to V 39	Home training (HT): 5 days/week-15 min/day<br>Speech therapy sessions (STS): 30 min/week	HT: 5 hr<br>STS: 2 hr
		V 40	Post-training 2B baseline assessment	10 min
	**TEST 4**	V 40	**Post-training assessment 2**	50 min
	**Break**	V 41, V 42	15 days without home training	1 hr
Phase 2	**INTENSIVE TRAINING 3 2 months**	V 43	Pre-training 3 baseline assessment	30 min
		V 44 to V 51	Home training (HT): 5 days/week-15 min/day<br>Speech therapy sessions (STS): 30 min/week	HT: 10 hr<br>STS: 4 hr
		V 52	Post-training 3 baseline assessment	10 min
	**TEST 5**	V 53-54	**Post-training assessment 3**	2 hr
Phase 3	**No intensive training 2 months**	V 56 to V 64	Stop home training<br>Speech therapy sessions (STS): 30 min/week	STS: 4 hr
	**TEST 6**	V 65	**Follow-up assessment**	15 min
Total		65 weeks	16 months	49 hr 20 min

PHASE 1 – TEST 2 – V 5 to V 14

SPEECH THERAPY SESSIONS (V 5 TO V 12)

For two months, the children are seen once a week for 30 minutes at the speech therapist's office, by the therapist who carried out the assessment. During these sessions, reading and spelling exercises are performed to compensate for the disorders. There is no daily home training during this phase.

	DATES (DD/MM/YYYY)	Reading	Spelling
V5	|__|__| / |__|__|/ |__|__|__|__|	❑	❑
V6	|__|__| / |__|__|/ |__|__|__|__|	❑	❑
V7	|__|__| / |__|__|/ |__|__|__|__|	❑	❑
V8	|__|__| / |__|__|/ |__|__|__|__|	❑	❑
V9	|__|__| / |__|__|/ |__|__|__|__|	❑	❑
V10	|__|__| / |__|__|/ |__|__|__|__|	❑	❑
V11	|__ |__| / |__|__|/ |__|__|__|__|	❑	❑
V12	|__|__| / |__|__|/ |__|__|__|__|	❑	❑

POST-CONTROL ASSESSMENT – V 13 – TEST 2 (PART 1)

For the administration and order of the tests, refer to the "testing manuals". Tests must be administered in a random order to be different for each participant.

Date: |__|__| / |__|__| / |__|__|__|__| ***(DD/MM/YYYY)***

- Reading meaningless text (Alouette©)
- Spelling (Chronosdictées© **version B**)
- Reading meaningful text: **Pingouin** (Evaléo© 6-15)
- Phonological analysis, non-word repetition, rapid automatized naming (Evalec©)
- Visuo-attentional span (Evadys©)
- Perception of the evolution of the reading disorder by the child and his parents evaluated by two questionnaires (Likert scales)

Internal Code	Patient Number	Group 1<br>Training PHONO1/VA2/IM3
19-HPNCL-02	|__|__| / |__|__| / |__|__|__|__|	

	Tests		Data	Raw scores	Rate + or -	Z Scores (SD or percentiles)	Time (min)
PHASE 1 TEST 2 V13	Reading meaningless text	"Alouette©"	Number of errors	|__|__|		|__|__|.|__|__|	3
			Number of words read	|__|__|__|		|__|__|.|__|__|	
			Number of correct words	|__|__|__|		|__|__|.|__|__|	
			Time in seconds	|__|__|__|		|__|__|.|__|__|	
			Accuracy index	|__|__|__|		|__|__|.|__|__|	
			Speed index	|__|__|__|		|__|__|.|__|__|	
			Reading age	|__|__| years |__|__| months			
	Spelling Test	"Chronosdictées©" Version B	Number of phonetic errors	|__|__|		|__|__|.|__|__|	10
			Number of lexical errors	|__|__|		|__|__|.|__|__|	
			Number of grammatical errors	|__|__|		|__|__|.|__|__|	
			Number of omissions	|__|__|		|__|__|.|__|__|	
			Number of segmentation errors	|__|__|		|__|__|.|__|__|	
			Total number of errors	|__|__|		|__|__|.|__|__|	
	Reading meaningful text "EVALEO©"	"Pingouin"	Number of words read	|__|__|__|		|__|	2
			Number of correct words	|__|__|__|		|__|	
			% correct/read	|__|__|__|.|__|		|__|	
			Degradation Index	|__|__|__|		|__|	
	Phonological awarness "EVALEC©"	Syllable suppression	Accuracy (% errors)	|__|__|__|.|__|		|__|__|.|__|__|	3
			Time in seconds	|__|__|__|.|__|__|		|__|__|.|__|__|	
		Phoneme suppression CVC	Accuracy (% errors)	|__|__|__|.|__|		|__|__|.|__|__|	
			Time in seconds	|__|__|__|.|__|__|		|__|__|.|__|__|	
		Phoneme suppression CCV	Accuracy (% errors)	|__|__|__|.|__|		|__|__|.|__|__|	
			Time in seconds	|__|__|__|.|__|__|		|__|__|.|__|__|	
		Non-word repetition	Span	|__|		|__|__|.|__|__|	1
			Time in seconds	|__|__|__|.|__|__|		|__|__|.|__|__|	
		Rapid Automatized Naming	Color recognition, %	|__|__|		|__|__|.|__|__|	2
			Color recognition, time in seconds	|__|__|__|.|__|__|		|__|__|.|__|__|	
			Reading names, %	|__|__|__|.|__|		|__|__|.|__|__|	
			Reading names, time in seconds	|__|__|__|.|__|__|		|__|__|.|__|__|	
	Visuo-attentional span "EVADYS"	Letter threshold	Threshold in ms	|__|__|__|		|__|__|.|__|__|	20
		Global Report GR	GR Score	|__|__|__|		|__|__|.|__|__|	
			GR Span	|__|.|__|__|		|__|__|.|__|__|	
		Partial Report PR	PR Score	|__|__|		|__|__|.|__|__|	
			PR Span	|__|.|__|__|		|__|__|.|__|__|	
	Surveys-Likert scale	Child pre-test	Total points	|__|__|			5
		Parents pre-test	Total points	|__|__|			
	Total time (min)						46

POST-CONTROL ASSESSMENT – V 14 – TEST 2 (PART 2)

For the administration and order of the tests, refer to the testing manuals.

Date: |__|__| / |__|__| / |__|__|__|__| ***(DD/MM/YYYY)***

- Global/local analysis (SIGL©)
- Reading regular, irregular words and non-words aloud (Evalec©)
- Short-term verbal memory and working memory, digit span (Evaléo©)
- Visuo-spatial span (Corsi©)
- Written comprehension (Orlec©)
- Reading meaningless text: DeltaText 2 (Bedoin©)
- Words read in 2 min, EVAL2M (Evaléo©)

<table>
<tr><th>Internal Code</th><th>Patient Number</th><th rowspan="2">Group 1
Training PHONO1/VA2/IM3</th></tr>
<tr><td>19-HPNCL-02</td><td>|__|__| / |__|__| / |__|__|__|__|</td></tr>
</table>

<table>
<tr><th rowspan="36">PHASE 1 TEST 2 V14</th><th colspan="2">Tests</th><th>Data</th><th>Raw scores</th><th>Rate + or -</th><th>Z Scores</th><th>Time (min)</th></tr>
<tr><td rowspan="6">Global/local analysis "SIGL© Drawings"</td><td rowspan="3">Response time (RT)</td><td>Local RT (ms)</td><td>|__|__|__|.|__|__|</td><td></td><td>|__|__|.|__|__|</td><td rowspan="6">20</td></tr>
<tr><td>Global RT (ms)</td><td>|__|__|__|.|__|__|</td><td></td><td>|__|__|.|__|__|</td></tr>
<tr><td>Interference asymmetry RT (ms)</td><td>|__|__|__|.|__|__|</td><td></td><td>|__|__|.|__|__|</td></tr>
<tr><td rowspan="3">Error Rate (ER)</td><td>Local ER (%)</td><td>|__|__|__|.|__|__|</td><td></td><td>|__|__|.|__|__|</td></tr>
<tr><td>Global ER (%)</td><td>|__|__|__|.|__|__|</td><td></td><td>|__|__|.|__|__|</td></tr>
<tr><td>Interference asymmetry ER (%)</td><td>|__|__|__|.|__|__|</td><td></td><td>|__|__|.|__|__|</td></tr>
<tr><td rowspan="14">Word reading "EVALEC©"</td><td rowspan="2">Regular words (RW)</td><td>Accuracy (% errors)</td><td>|__|__|__|.|__|__|</td><td></td><td>|__|__|.|__|__|</td><td rowspan="6">8</td></tr>
<tr><td>Time in ms</td><td>|__|__|__|__|</td><td></td><td>|__|__|.|__|__|</td></tr>
<tr><td rowspan="2">Irregular words (IW)</td><td>Accuracy (% errors)</td><td>|__|__|__|.|__|__|</td><td></td><td>|__|__|.|__|__|</td></tr>
<tr><td>Time in ms</td><td>|__|__|__|__|</td><td></td><td>|__|__|.|__|__|</td></tr>
<tr><td rowspan="2">Non-words (NW)</td><td>Accuracy (% errors)</td><td>|__|__|__|.|__|__|</td><td></td><td>|__|__|.|__|__|</td></tr>
<tr><td>Time in ms</td><td>|__|__|__|__|</td><td></td><td>|__|__|.|__|__|</td></tr>
<tr><td rowspan="2">Short IW</td><td>Accuracy (% errors)</td><td>|__|__|__|.|__|__|</td><td></td><td>|__|__|.|__|__|</td><td></td></tr>
<tr><td>Time in ms</td><td>|__|__|__|__|</td><td></td><td>|__|__|.|__|__|</td><td></td></tr>
<tr><td rowspan="2">Long IW</td><td>Accuracy (% errors)</td><td>|__|__|__|.|__|__|</td><td></td><td>|__|__|.|__|__|</td><td></td></tr>
<tr><td>Time in ms</td><td>|__|__|__|__|</td><td></td><td>|__|__|.|__|__|</td><td></td></tr>
<tr><td rowspan="2">Short NW</td><td>Accuracy (% errors)</td><td>|__|__|__|.|__|__|</td><td></td><td>|__|__|.|__|__|</td><td></td></tr>
<tr><td>Time in ms</td><td>|__|__|__|__|</td><td></td><td>|__|__|.|__|__|</td><td></td></tr>
<tr><td rowspan="2">Long NW</td><td>Accuracy (% errors)</td><td>|__|__|__|.|__|__|</td><td></td><td>|__|__|.|__|__|</td><td rowspan="2">5</td></tr>
<tr><td>Time in ms</td><td>|__|__|__|__|</td><td></td><td>|__|__|.|__|__|</td></tr>
<tr><td rowspan="4">Digit span</td><td rowspan="4">"EVALEO©"</td><td>Score forward span</td><td>|__|</td><td></td><td>|__|</td><td rowspan="4">8</td></tr>
<tr><td>Forward span</td><td>|__|</td><td></td><td>|__|</td></tr>
<tr><td>Score backward span</td><td>|__|</td><td></td><td>|__|</td></tr>
<tr><td>Backward span</td><td>|__|</td><td></td><td>|__|</td></tr>
<tr><td rowspan="2">Visuo-spatial span</td><td rowspan="2">"Corsi©"</td><td>Forward span</td><td>|__|</td><td></td><td>|__|__|.|__|__|</td><td rowspan="2">8</td></tr>
<tr><td>Backward span</td><td>|__|</td><td></td><td>|__|__|.|__|__|</td></tr>
<tr><td rowspan="2">Written comprehension</td><td rowspan="2">"Orlec©, L3"</td><td>Number of correct items</td><td>|__|__|</td><td></td><td>|__|__|.|__|__|</td><td rowspan="2">5</td></tr>
<tr><td>Percentage of correct items</td><td>|__|__|__|.|__|__|</td><td></td><td>|__|__|.|__|__|</td></tr>
<tr><td rowspan="3">Reading meaningless text</td><td rowspan="3">"DeltaText© 2"</td><td>Number of words read</td><td>|__|__|__|</td><td></td><td></td><td rowspan="3">3</td></tr>
<tr><td>Number of errors</td><td>|__|__|__|</td><td></td><td></td></tr>
<tr><td>Time in seconds</td><td>|__|__|__|</td><td></td><td></td></tr>
<tr><td rowspan="3">Words read in 2 min</td><td rowspan="3">"Eval 2M©"</td><td>Number of words read</td><td>|__|__|__|</td><td></td><td>|__|</td><td rowspan="3">2</td></tr>
<tr><td>Number of correct words</td><td>|__|__|__|</td><td></td><td>|__|</td></tr>
<tr><td>% correct/read</td><td>|__|__|__|.|__|__|</td><td></td><td>|__|</td></tr>
<tr><td colspan="6">Total time (min)</td><td>59</td></tr>
</table>

PHASE 2 – PHONO 1 TRAINING – TEST 3 – V 15 to V 26

PRE-TRAINING PHONO 1A BASELINE ASSESSMENT, INSTRUCTIONS, V 15

Date: |__|__| / |__|__| / |__|__|__|__| ***(DD/MM/YYYY)***

If the participant has been randomized to Group 1, she/he begins with phonological training. Phonological training lasts two months and must be adapted by the clinician to the participant's cognitive profile based on the results obtained in the Evalec test (phonological analysis, non-word repetition, rapid automated naming) and Rapdys assessment.

For one month, the child will complete Phono 1A training if she/he obtains a score of less than -1.5 SD on at least one
Evalec test and a score of less than 70% in identification and/or discrimination in the Rapdys evaluation test.
The exercises target phoneme fusion and categorical perception. Before starting the training, administer the following baseline tests and report the results in the table below.
NB: If the participant does not have a categorical perceptual disorder, complete Phono 1B training.

**PHONO 1A training – first month – V 15**			**Time**
Pre-training PHONO 1A baseline		Raw scores	
BSL 1 Items seen	Phoneme fusion, accuracy out of 10	|__|__|	2
	Phoneme fusion, time in seconds	|__|__|__|	
	Rapdys, identification, %	|__|__|__|	5
	Rapdys, discrimination, %	|__|__|__|	
BSL 2 Items not seen	Phoneme fusion, accuracy out of 10	|__|__|	2
	Phoneme fusion, time in seconds	|__|__|__|	
	Spoonerism (Evaléo) accuracy	|__|	5
	Spoonerism (Evaléo) time	|__|__|__|.|__|__|	
BSL 3	Reading non-words, score out of 20	|__|__|	1
	Reading non-words, time in seconds	|__|__|__|.|__|__|	
BSL 4	Fluidity drawings in 1 min	|__|__|	1
Total time			16

Give instructions to parents and children on how to complete 1A ***phonological training*** for 1 month at home (15 minutes a day, 5 days a week). See group 1 instruction manual.

SPEECH THERAPY SESSIONS (V 16 TO V 19)

During the home training phase, the children are trained once a week for 30 minutes by the speech therapist, at the latter's office. They complete their exercises for 15 minutes with the speech therapist who checks the smooth running of the training. The rest of the session is focused on reading and spelling exercises

	DATES (DD/MM/YYYY)	Reading	Spelling	Training
V16	I__I__I / I__I__I/ I__I__I__I__I	❑	❑	❑
V17	I__I__I / I__I__I/ I__I__I__I__I	❑	❑	❑
V18	I__I__I / I__I__I/ I__I__I__I__I	❑	❑	❑
V19	I__I__I / I__I__I/ I__I__I__I__I	❑	❑	❑

POST-TRAINING PHONO 1A BASELINE ASSESSMENT, V 20

After one month of 1A phonological training, administer the same post-training baseline tests and report the results in the

table below.

PHONO 1A training – first month – V 20			Time
Post-training PHONO 1A baseline		Raw scores	
BSL 1 Items seen	Phoneme fusion, accuracy out of 10	|__|__|	2
	Phoneme fusion, time in seconds	|__|__|__|	
	Rapdys, identification, %	|__|__|__|	5
	RapDys, discrimination, %	|__|__|__|	
BSL 2 Items not seen	Phoneme fusion, accuracy out of 10	|__|__|	2
	Phoneme fusion, time in seconds	|__|__|__|	
	Spoonerism (Evaléo) accuracy	|__|	5
	Spoonerism (Evaléo) time	|__|__|__|__|.|__|__|__|	
BSL 3	Reading non-word, score out of 20	|__|__|	1
	Reading non-word, time in seconds	|__|__|__|__|.|__|__|__|	
BSL 4	Fluidity drawings in 1 min	|__|__|	1
Total time			16

PRE-TRAINING PHONO 1B BASELINE ASSESSMENT, SECOND MONTH

Date: |__|__| / |__|__| / |__|__|__|__| ***(DD/MM/YYYY)***

After the first month of training, further exercises of the **1B phonological training** program are prescribed the second month if the participant achieves a score of less than 1.5 standard deviation in at least one Evalec test.

The exercises target phoneme segmentation and phonological memory.

Before starting training, complete the following baseline tests and report the results in the table below.

NB: Note: If the participant does not have a phonological short-term memory impairment, refer to the CRF G1 RAN/VA/CM for rapid automatized naming training.

PHONO 1B training – second month – V 21			Time
Pre-training PHONO 1B baseline		Raw scores	
BSL 1 Items seen	Phoneme segmentation, accuracy out of 10	|__|__|	2
	Segmentation of phonemes, time in seconds	|__|__|__|	
	Comparisons of 5 sound sequences (Phonopidow)	|__|__|	2
BSL 2 Items not seen	Phoneme segmentation, accuracy out of 10	|__|__|	2
	Segmentation of phonemes, time in seconds	|__|__|__|	
	Hearing acronyms (Belec), accuracy	|__|__|	2
	Hearing acronyms (Belec), time	|__|__|__| . |__|__|	
BSL 3	Reading non-word, score out of 20	|__|__|	1
	Reading non-word, time in seconds	|__|__|__| . |__|__|	
BSL 4	Mathematical symbols, items completed in 1 min	|__|__|	1
Total time			10

Give instructions to parents and children on how to perform 1B phonological training for 1 month at home (15 minutes

a day, 5 days a week). See group 1 instruction manual.

SPEECH THERAPY SESSIONS (V 22 TO V 25)

During home training, the children are seen by the speech therapist once a week for 30 minutes at the speech therapist's office. They complete their exercises for 15 minutes with the speech therapist who checks the smooth running of the training. The rest of the session is focused on reading and spelling exercises (i.e. the automation of grapheme/phoneme matches for graphemes that have not yet been acquired, such as syllabic impregnation, Borel gestures, etc.).

	DATES (DD/MM/YYYY)	Reading	Spelling	Training
V22	|__|__| / |__|__|/ |__|__|__|__|	❑	❑	❑
V23	|__|__| / |__|__|/ |__|__|__|__|	❑	❑	❑
V24	|__|__| / |__|__|/ |__|__|__|__|	❑	❑	❑
V25	|__|__| / |__|__|/ |__|__|__|__|	❑	❑	❑

POST-TRAINING PHONO 1B BASELINE ASSESSMENT, V 26

Date: |__|__| / |__|__| / |__|__|__|__| (DD/MM/YYYY)

After one month of training, administer the same post-training PHONO 1B baseline tests and report the results in the table below.

PHONO 1B training – second month – V 26			Time
Post-training PHONO 1B baseline		Raw scores	
BSL 1<br>Items seen	Phoneme segmentation, accuracy out of 10	|__|__|	2
	Segmentation of phonemes, time in seconds	|__|__|__|	
	Comparison of 5 sound sequences (Phonopidow)	|__|__|	2
BSL 2<br>Items not seen	Phoneme segmentation, accuracy out of 10	|__|__|	2
	Segmentation of phonemes, time in seconds	|__|__|__|	
	Hearing acronyms (Belec), accuracy	|__|__|	2
	Hearing acronyms (Belec), time	|__|__|__| . |__|__|	
BSL 3	Reading non-words, score out of 20	|__|__|	1
	Reading non-words, time in seconds	|__|__|__| . |__|__|	
BSL 4	Mathematical symbols, items completed in 1 min	|__|__|	1
Total time			10

POST-TRAINING ASSESSMENT 1 – V26 – TEST 3

For the administration and order of the tests, refer to the "testing manuals". Tests must be administered in a random order to be different for each participant.

During the same session, V 26, administer Test 3 at the end of the two months of phonological training 1.

- Reading meaningless text, DeltaText© 3 (Bedoin)
- Reading meaningless text, Alouette©
- Reading meaningful text, **La Mouette** (Evaléo© 6-15)
- Words read in 2 min, EVAL2M (Evaléo© 6-15)
- Attentional focus local/global (SIGL©)
- Phonological analysis, non-word repetition, rapid automatized naming (Evalec©)
- Visuo-attentional span (Evadys©)

<table>
<tr><th>Internal Code</th><th>Patient Number</th><th rowspan="2">Group 1
Training PHONO1/VA2/IM3</th></tr>
<tr><td>19-HPNCL-02</td><td>|__|__| / |__|__| / |__|__|__|__|</td></tr>
</table>

<table>
<tr><th></th><th colspan="2">Tests</th><th>Data</th><th>Raw scores</th><th>Rate + or -</th><th>Z Scores (SD or percentiles)</th><th>Time (min)</th></tr>
<tr><td rowspan="40">PHASE 2 TEST 3 V26</td><td rowspan="7">Reading meaningless text</td><td rowspan="7">"Alouette©"</td><td>Number of errors</td><td>|__|__|</td><td></td><td>|__|__|.|__|__|</td><td rowspan="7">3</td></tr>
<tr><td>Number of words read</td><td>|__|__|__|</td><td></td><td>|__|__|.|__|__|</td></tr>
<tr><td>Number of correct words</td><td>|__|__|__|</td><td></td><td>|__|__|.|__|__|</td></tr>
<tr><td>Time in seconds</td><td>|__|__|__|</td><td></td><td>|__|__|.|__|__|</td></tr>
<tr><td>Accuracy index</td><td>|__|__|__|</td><td></td><td>|__|__|.|__|__|</td></tr>
<tr><td>Speed index</td><td>|__|__|__|</td><td></td><td>|__|__|.|__|__|</td></tr>
<tr><td>Reading age</td><td colspan="3">|__|__| years |__|__| months</td></tr>
<tr><td rowspan="3">Reading meaningless text</td><td rowspan="3">"DeltaText© 2"</td><td>Number of words read</td><td>|__|__|__|</td><td></td><td></td><td rowspan="3">3</td></tr>
<tr><td>Number of errors</td><td>|__|__|__|</td><td></td><td></td></tr>
<tr><td>Time in seconds</td><td>|__|__|__|</td><td></td><td></td></tr>
<tr><td rowspan="3">Words read in 2 min</td><td rowspan="3">"Eval 2M©"</td><td>Number of words read</td><td>|__|__|__|</td><td></td><td>|__|</td><td rowspan="3">2</td></tr>
<tr><td>Number of correct words</td><td>|__|__|__|</td><td></td><td>|__|</td></tr>
<tr><td>% correct/read</td><td>|__|__|__|.|__|__|</td><td></td><td>|__|</td></tr>
<tr><td rowspan="4">Reading meaningful text "EVALEO©"</td><td rowspan="4">"Mouette"</td><td>Number of words read</td><td>|__|__|__|</td><td></td><td>|__|</td><td rowspan="4">2</td></tr>
<tr><td>Number of correct words</td><td>|__|__|__|</td><td></td><td>|__|</td></tr>
<tr><td>% correct/read</td><td>|__|__|__|.|__|</td><td></td><td>|__|</td></tr>
<tr><td>Degradation Index</td><td>|__|__|__|</td><td></td><td>|__|</td></tr>
<tr><td rowspan="6">Global/local analysis "SIGL© Drawings"</td><td rowspan="3">Response time (RT)</td><td>Local RT (ms)</td><td>|__|__|__|.|__|__|</td><td></td><td>|__|__|.|__|__|</td><td rowspan="6">20</td></tr>
<tr><td>Global RT (ms)</td><td>|__|__|__|.|__|__|</td><td></td><td>|__|__|.|__|__|</td></tr>
<tr><td>Interference asymmetry RT (ms)</td><td>|__|__|__|.|__|__|</td><td></td><td>|__|__|.|__|__|</td></tr>
<tr><td rowspan="3">Error Rate (ER)</td><td>Local ER (%)</td><td>|__|__|__|.|__|__|</td><td></td><td>|__|__|.|__|__|</td></tr>
<tr><td>Global ER (%)</td><td>|__|__|__|.|__|__|</td><td></td><td>|__|__|.|__|__|</td></tr>
<tr><td>Interference asymmetry ER (%)</td><td>|__|__|__|.|__|__|</td><td></td><td>|__|__|.|__|__|</td></tr>
<tr><td rowspan="5">Visuo-attentional span "EVADYS©"</td><td>Letter threshold</td><td>Threshold in ms</td><td>|__|__|__|</td><td></td><td>|__|__|.|__|__|</td><td rowspan="5">20</td></tr>
<tr><td rowspan="2">Global Report (GR)</td><td>GR Score</td><td>|__|__|__|</td><td></td><td>|__|__|.|__|__|</td></tr>
<tr><td>GR Span</td><td>|__|.|__|__|</td><td></td><td>|__|__|.|__|__|</td></tr>
<tr><td rowspan="2">Partial Report (PR)</td><td>PR Score</td><td>|__|__|</td><td></td><td>|__|__|.|__|__|</td></tr>
<tr><td>PR Span</td><td>|__|.|__|__|</td><td></td><td>|__|__|.|__|__|</td></tr>
<tr><td rowspan="12">Phonological awarness "EVALEC©"</td><td rowspan="2">Syllable suppression</td><td>Accuracy (% errors)</td><td>|__|__|__|.|__|</td><td></td><td>|__|__|.|__|__|</td><td rowspan="6">3</td></tr>
<tr><td>Time in seconds</td><td>|__|__|__|.|__|__|</td><td></td><td>|__|__|.|__|__|</td></tr>
<tr><td rowspan="2">Phoneme Suppression CVC</td><td>Accuracy (% errors)</td><td>|__|__|__|.|__|</td><td></td><td>|__|__|.|__|__|</td></tr>
<tr><td>Time in seconds</td><td>|__|__|__|.|__|__|</td><td></td><td>|__|__|.|__|__|</td></tr>
<tr><td rowspan="2">Phoneme suppression CCV</td><td>Accuracy (% errors)</td><td>|__|__|__|.|__|</td><td></td><td>|__|__|.|__|__|</td></tr>
<tr><td>Time in seconds</td><td>|__|__|__|.|__|__|</td><td></td><td>|__|__|.|__|__|</td></tr>
<tr><td rowspan="2">Non-words repetition</td><td>Span</td><td>|__|</td><td></td><td>|__|__|.|__|__|</td><td rowspan="2">1</td></tr>
<tr><td>Time in seconds</td><td>|__|__|__|.|__|__|</td><td></td><td>|__|__|.|__|__|</td></tr>
<tr><td rowspan="4">Rapid Automatized Naming</td><td>Color recognition, %</td><td>|__|__|</td><td></td><td>|__|__|.|__|__|</td><td rowspan="4">2</td></tr>
<tr><td>Color recognition, time in seconds</td><td>|__|__|__|.|__|__|</td><td></td><td>|__|__|.|__|__|</td></tr>
<tr><td>Reading names, %</td><td>|__|__|__|.|__|</td><td></td><td>|__|__|.|__|__|</td></tr>
<tr><td>Reading names, time in seconds</td><td>|__|__|__|.|__|__|</td><td></td><td>|__|__|.|__|__|</td></tr>
<tr><td></td><td colspan="6">Total time (min)</td><td>51</td></tr>
</table>

15-DAY BREAK WITHOUT TRAINING

A 15-day break without training is provided before moving on to 2 periods of visuo-attentional training. Weekly sessions continue at the office if the participant is not on vacation. In this case, please indicate it in the "comments."

	DATES (DD/MM/YYYY)	Reading	Spelling	Comments
V27	|__|__| / |__|__|/ |__|__|__|__|	❑	❑	
V28	|__|__| / |__|__|/ |__|__|__|__|	❑	❑	

PHASE 2 – VA 2 TRAINING – TEST 4 – V 29 to V 40

PRE-TRAINING VA 2A BASELINE ASSESSMENT– INSTRUCTIONS

Date: |__|__| / |__|__| / |__|__|__|__| ***(DD/MM/YYYY)***

After phonological training 1, the participants complete visuo-attentional training 2 for two months.

VA training *must* be adapted by the investigator to the participant's cognitive profile based on the results obtained on the Evadys test (global report and partial report) and SIGL (interference asymmetry, TE and TR).

For one month, the child will complete VA 2A training if she/he achieves a score of less than 1.5 standard deviation on at least one Evadys test. The exercises target the visuo-attentional span and spelling memory.

Before you start training, administer the following baseline tests, and report the results in the table below.

Internal Code	Patient Number	Group 1<br>Training PHONO1/VA2/IM3
19-HPNCL-02	|__|__| / |__|__| / |__|__|__|__|	

VA 2A training – first month – V 29			Time
Pre-training VA 2A baseline		Raw scores	
BSL 1	Maeva, score out of 10 (accuracy)	|__|__|	1
BSL 2	VA global span numbers (Evaleo)	|__| . |__|__|	4
	RC, EVA, out of 100	|__|__|__|	
BSL 3	Reading irregular words, score out of 20	|__|__|	1
	Reading irregular words, time in seconds	|__|__|__| . |__|__|	
BSL 4	Codes, time in seconds	|__|__|__|	1
Total time			7

Then give instructions to parents and the child on how to complete the visuo-attentional training 2A for 1 month at home (15 minutes a day, 5 days a week). See the “testing manual” for instructions.

SPEECH THERAPY SESSIONS (V 30 TO V 33)

During home training, children are seen by the speech therapist once a week for 30 minutes at the speech therapist's office. They complete their exercises for 15 minutes with the speech therapist who checks the smooth running of the training. The rest of the session focuses on reading and spelling exercises (i.e. reading and transcribing graphemes).

	DATES (DD/MM/YYYY)	Reading	Spelling	Training
V30	|__|__| / |__|__|/ |__|__|__|__|	❑	❑	❑
V31	|__|__| / |__|__|/ |__|__|__|__|	❑	❑	❑
V32	|__|__| / |__|__|/ |__|__|__|__|	❑	❑	❑
V33	|__|__| / |__|__|/ |__|__|__|__|	❑	❑	❑

POST-TRAINING VA 2A BASELINE ASSESSMENT, V 34

After one month of training, administer the same post-training baseline tests and report the results in the table below.

VA 2A training – first month – V 34			Time
Post-training VA 2A baseline		Raw scores	
BSL 1	Maeva, score out of 10 (accuracy)	|__|__|	1
BSL 2	VA global span numbers (Evaleo)	|__| . |__|__|	4
	RC, EVA, out of 100	|__|__|__|	
BSL 3	Reading irregular words, score out of 20	|__|__|	1
	Reading irregular words, time in seconds	|__|__|__| . |__|__|	
BSL 4	Codes, time in seconds	|__|__|__|	1
Total Time			7

PRE-TRAINING VA 2B BASELINE ASSESSMENT – SECOND MONTH – INSTRUCTIONS – V35

Date : |__|__| / |__|__| / |__|__|__|__| ***(DD/MM/YYYY)***

After one first month of training, other exercises are given the second month. Before you start training, administer the following baseline tests, and report the results in the table below.

VA 2B training – second month – V 35				Time
Pre-training VA 2B baseline			Raw scores	
BSL1	Complex triplets Switchipido	Good answer scores	|__|__|	2
		Wrong answer scores	|__|__|	
		Average time in ms	|__|__|__|__|	
BSL2	VA global span numbers, Evaléo		|__| . |__|__|	4
	RC, EVA, out of 100		|__|__|__|	
BSL3	Reading irregular words, score out of 20		|__|__|	1
	Reading irregular words, time in seconds		|__|__|__| . |__|__|	
BSL4	Mental calculation, items completed in 2 minutes		|__|__|	1
Total time				7

SPEECH THERAPY SESSIONS (V 36 TO V 39)

During home training, children are seen by the speech therapist once a week for 30 minutes in the speech therapist's office.

	DATES (DD/MM/YYYY)	Reading	Spelling	Training
V36	|__|__| / |__|__|/ |__|__|__|__|	❑	❑	❑
V37	|__|__| / |__|__|/ |__|__|__|__|	❑	❑	❑
V38	|__|__| / |__|__|/ |__|__|__|__|	❑	❑	❑
V39	|__|__| / |__|__|/ |__|__|__|__|	❑	❑	❑

POST-TRAINING VA 2B BASELINE ASSESSMENT- TEST 4 – V 40

Date: |__|__| / |__|__| / |__|__|__|__| (DD/MM/YYYY)

After a month of training, administer the same post-training baseline tests and report the results in the table below.

VA 2B training – second month – V 40				Time
Post-training VA 2B baseline			Raw scores	
BSL1	Complex triplets Switchipido	Good answer scores	|__|__|	2
		Wrong answer scores	|__|__|	
		Average time in ms	|__|__|__|__|	
BSL2	VA global span numbers, Evaléo		|__| . |__|__|	4
	RC, EVA, out of 100		|__|__|__|	
BSL3	Reading irregular words, score out of 20		|__|__|	1
	Reading irregular words, time in seconds		|__|__|__| . |__|__|	
BSL4	Mental calculation, items completed in 2 minutes		|__|__|	1
Total time				7

POST TRAINING ASSESSMENT 2 – V 40 – TEST 4

For the administration of the tests, refer to the "testing manuals". Tests must be administered in a random order to be different for each participant.

During the same session, V 40, perform Test 4 at the end of the 2 months of visuo-attentional training.

- DeltaText© 4
- Alouette©
- Pingouin (Evaléo© 6-15)
- EVAL2M (Evaléo© 6-15)

<table>
<tr><th>Internal Code</th><th>Patient Number</th><th rowspan="2">Group 1
Training PHONO1/VA2/IM3</th></tr>
<tr><td>19-HPNCL-02</td><td>|__|__| / |__|__| / |__|__|__|__|</td></tr>
</table>

<table>
<tr><td rowspan="16">PHASE 2 TEST 4 V40</td><th colspan="2">Tests</th><th>Data</th><th>Raw scores</th><th>Rate + or -</th><th>Z Scores (SD or percentiles)</th><th>Time (min)</th></tr>
<tr><td rowspan="7">Reading meaningless text</td><td rowspan="7">"Alouette©"</td><td>Number of errors</td><td>|__|__|</td><td></td><td>|__|__|.|__|__|</td><td rowspan="7">3</td></tr>
<tr><td>Number of words read</td><td>|__|__|__|</td><td></td><td>|__|__|.|__|__|</td></tr>
<tr><td>Number of correct words</td><td>|__|__|__|</td><td></td><td>|__|__|.|__|__|</td></tr>
<tr><td>Time in seconds</td><td>|__|__|__|</td><td></td><td>|__|__|.|__|__|</td></tr>
<tr><td>Accuracy index</td><td>|__|__|__|</td><td></td><td>|__|__|.|__|__|</td></tr>
<tr><td>Speed index</td><td>|__|__|__|</td><td></td><td>|__|__|.|__|__|</td></tr>
<tr><td>Reading age</td><td colspan="3">|__|__| years |__|__| months</td></tr>
<tr><td rowspan="3">Reading meaningless text</td><td rowspan="3">"DeltaText© 2"</td><td>Number of words read</td><td>|__|__|__|</td><td></td><td></td><td rowspan="3">3</td></tr>
<tr><td>Number of errors</td><td>|__|__|__|</td><td></td><td></td></tr>
<tr><td>Time in second</td><td>|__|__|__|</td><td></td><td></td></tr>
<tr><td rowspan="3">Words read in 2 min</td><td rowspan="3">"Eval 2M©"</td><td>Number of words read</td><td>|__|__|__|</td><td></td><td>|__|</td><td rowspan="3">2</td></tr>
<tr><td>Number of correct words</td><td>|__|__|__|</td><td></td><td>|__|</td></tr>
<tr><td>% correct/read</td><td>|__|__|__|.|__|__|</td><td></td><td>|__|</td></tr>
<tr><td rowspan="4">Reading meaningful text "EVALEO©"</td><td rowspan="4">"Pingouin"</td><td>Number of words read</td><td>|__|__|__|</td><td></td><td>|__|</td><td rowspan="4">2</td></tr>
<tr><td>Number of correct words</td><td>|__|__|__|</td><td></td><td>|__|</td></tr>
<tr><td></td><td></td><td>% correct/read</td><td>|__|__|__|.|__|</td><td></td><td>|__|</td></tr>
<tr><td></td><td></td><td></td><td>Degradation Index</td><td>|__|__|__|</td><td></td><td>|__|</td></tr>
<tr><td></td><td colspan="6">Total time (min)</td><td>10</td></tr>
</table>

Internal Code	Patient Number	Group 1<br>Training PHONO1/VA2/IM3
19-HPNCL-02	|__|__| / |__|__| / |__|__|__|__|	

15-DAY BREAK WITHOUT TRAINING

A 15-day break without training is provided before moving on to cross-modal training. During this break, weekly STS continue if the participant is not on vacation. In this case, please indicate it in the "comments."

	DATES (DD/MM/YYYY)	Reading	Spelling	Comments
V41	|__|__| / |__|__|/ |__|__|__|__|	❑	❑	
V42	|__|__| / |__|__|/ |__|__|__|__|	❑	❑	

PHASE 3 – CM 3 TRAINING – TEST 5 – V 43 to V 55

PRE-TRAINING IM 3 BASELINE ASSESSMENT – INSTRUCTIONS – V 43

Date : |__|__| / |__|__| / |__|__|__|__| ***(DD/MM/YYYY)***

After the two periods of *phonological training* and the two periods of *visuo-attentional training*, the participant completes the third and final training period which is *cross-modal training* for two months. All participants (groups 1 and 2) have the same training.

CM 3A training – V 43			Time
Pre-training IM 3 baseline		Raw scores	
BSL1	Reading 20 words seen, accuracy	|__|__|	1
	Reading 20 words seen, time in seconds	|__|__| . |__|__|	1
BSL2	Transcribing 10 words seen	|__|__|	2
BSL3	Reading regular words, accuracy	|__|__|	1
	Reading regular words, time in seconds	|__|__| . |__|__|	
BSL4	Labyrinth, time in seconds	|__|__| . |__|__|	1
Total Time			6

Give instructions to parents and children on how to perform cross-modal training 3 with GraphoGame (10 *min* per day) and ARMAR training (5 minutes per day) for 2 months, 5 days/week. See the instruction book.

SPEECH THERAPY SESSION (V44 TO V 51)

During home training, children are seen by the speech therapist once a week for 30 minutes in the speech therapist's office.

	DATES (DD/MM/YYYY)	Reading	Spelling	Training
V44	|__|__| / |__|__|/ |__|__|__|__|	❑	❑	❑
V45	|__|__| / |__|__|/ |__|__|__|__|	❑	❑	❑
V46	|__|__| / |__|__|/ |__|__|__|__|	❑	❑	❑
V47	|__|__| / |__|__|/ |__|__|__|__|	❑	❑	❑

	DATES (DD/MM/YYYY)	Reading	Spelling	Training
V48	|__|__| / |__|__|/ |__|__|__|__|	❑	❑	❑
V49	|__|__| / |__|__|/ |__|__|__|__|	❑	❑	❑
V50	|__|__| / |__|__|/ |__|__|__|__|	❑	❑	❑
V51	|__|__| / |__|__|/ |__|__|__|__|	❑	❑	❑

POST-TRAINING IM 3 BASELINE ASSESSMENT – V 51

Date : |__|__| / |__|__| / |__|__|__|__| ***(DD/MM/YYYY)***

After two months of training, perform the same post-training baseline tests and report the results in the table below.

CM 3A training – V 43			Time
Post-training IM 3 baseline		Raw scores	
BSL1	Reading 20 words seen, accuracy	|__|__|	1
	Reading 20 words seen, time in seconds	|__|__| . |__|__|	1
BSL2	Transcription of 10 words seen	|__|__|	2
BSL3	Reading regular words, accuracy	|__|__|	1
	Reading regular words, time in seconds	|__|__| . |__|__|	
BSL4	Labyrinth, time in seconds	|__|__| . |__|__|	1
Total Time			6

POST TRAINING ASSESSMENT 3 – TEST 5 – V52 AND V53

For the administration and order of the tests, refer to the "testing manuals". Tests must be administered in a random order to be different for each participant.

Date : |__|__| / |__|__| / |__|__|__|__| ***(DD/MM/YYYY)***

Same assessment as test 2.

<table>
<tr><th>Internal Code</th><th>Patient Number</th><th rowspan="2">Group 1
Training PHONO1/VA2/IM3</th></tr>
<tr><td>19-HPNCL-02</td><td>|__|__| / |__|__| / |__|__|__|__|</td></tr>
</table>

<table>
<tr><td rowspan="36">PHASE 2 TEST 5 V52</td><th colspan="2">Tests</th><th>Data</th><th>Raw scores</th><th>Rate + or -</th><th>Z Scores (SD or percentiles)</th><th>Time (min)</th></tr>
<tr><td rowspan="7">Reading meaningless text</td><td rowspan="7">"Alouette©"</td><td>Number of errors</td><td>|__|__|</td><td></td><td>|__|__|.|__|__|</td><td rowspan="7">3</td></tr>
<tr><td>Number of words read</td><td>|__|__|__|</td><td></td><td>|__|__|.|__|__|</td></tr>
<tr><td>Number of correct words</td><td>|__|__|__|</td><td></td><td>|__|__|.|__|__|</td></tr>
<tr><td>Time in seconds</td><td>|__|__|__|</td><td></td><td>|__|__|.|__|__|</td></tr>
<tr><td>Accuracy index</td><td>|__|__|__|</td><td></td><td>|__|__|.|__|__|</td></tr>
<tr><td>Speed index</td><td>|__|__|__|</td><td></td><td>|__|__|.|__|__|</td></tr>
<tr><td>Reading age</td><td colspan="3">|__|__| years |__|__| months</td></tr>
<tr><td rowspan="6">Spelling Test</td><td rowspan="6">"Chronosdictées©" Version A</td><td>Number of phonetic errors</td><td>|__|__|</td><td></td><td>|__|__|.|__|__|</td><td rowspan="6">10</td></tr>
<tr><td>Number of lexical errors</td><td>|__|__|</td><td></td><td>|__|__|.|__|__|</td></tr>
<tr><td>Number of grammatical errors</td><td>|__|__|</td><td></td><td>|__|__|.|__|__|</td></tr>
<tr><td>Number of omissions</td><td>|__|__|</td><td></td><td>|__|__|.|__|__|</td></tr>
<tr><td>Number of segmentation errors</td><td>|__|__|</td><td></td><td>|__|__|.|__|__|</td></tr>
<tr><td>Total number of errors</td><td>|__|__|</td><td></td><td>|__|__|.|__|__|</td></tr>
<tr><td rowspan="4">Reading meaningful text "EVALEO©"</td><td rowspan="4">"Mouette"</td><td>Number of words read</td><td>|__|__|__|</td><td></td><td>|__|</td><td rowspan="4">2</td></tr>
<tr><td>Number of correct words</td><td>|__|__|__|</td><td></td><td>|__|</td></tr>
<tr><td>% correct/read</td><td>|__|__|__|.|__|</td><td></td><td>|__|</td></tr>
<tr><td>Degradation Index</td><td>|__|__|__|</td><td></td><td>|__|</td></tr>
<tr><td rowspan="12">Phonological awarness "EVALEC©"</td><td rowspan="2">Syllable suppression</td><td>Accuracy (% errors)</td><td>|__|__|__|.|__|</td><td></td><td>|__|__|.|__|__|</td><td rowspan="6">3</td></tr>
<tr><td>Time in seconds</td><td>|__|__|__|.|__|__|</td><td></td><td>|__|__|.|__|__|</td></tr>
<tr><td rowspan="2">Phoneme suppression CVC</td><td>Accuracy (% errors)</td><td>|__|__|__|.|__|</td><td></td><td>|__|__|.|__|__|</td></tr>
<tr><td>Time in seconds</td><td>|__|__|__|.|__|__|</td><td></td><td>|__|__|.|__|__|</td></tr>
<tr><td rowspan="2">Phoneme suppression CCV</td><td>Accuracy (% errors)</td><td>|__|__|__|.|__|</td><td></td><td>|__|__|.|__|__|</td></tr>
<tr><td>Time in seconds</td><td>|__|__|__|.|__|__|</td><td></td><td>|__|__|.|__|__|</td></tr>
<tr><td rowspan="2">Non-word repetition</td><td>Span</td><td>|__|</td><td></td><td>|__|__|.|__|__|</td><td rowspan="2">1</td></tr>
<tr><td>Time in seconds</td><td>|__|__|__|.|__|__|</td><td></td><td>|__|__|.|__|__|</td></tr>
<tr><td rowspan="4">Rapid Automatized Naming</td><td>Color recognition, %</td><td>|__|__|</td><td></td><td>|__|__|.|__|__|</td><td rowspan="4">2</td></tr>
<tr><td>Color recognition, time in seconds</td><td>|__|__|__|.|__|__|</td><td></td><td>|__|__|.|__|__|</td></tr>
<tr><td>Reading names, %</td><td>|__|__|__|.|__|</td><td></td><td>|__|__|.|__|__|</td></tr>
<tr><td>Reading names, time in seconds</td><td>|__|__|__|.|__|__|</td><td></td><td>|__|__|.|__|__|</td></tr>
<tr><td rowspan="5">Visuo-attentional span "EVADYS©"</td><td>Letter threshold</td><td>Threshold in ms</td><td>|__|__|__|</td><td></td><td>|__|__|.|__|__|</td><td rowspan="5">20</td></tr>
<tr><td rowspan="2">Global Report (GR)</td><td>GR Score</td><td>|__|__|__|</td><td></td><td>|__|__|.|__|__|</td></tr>
<tr><td>GR Span</td><td>|__|.|__|__|</td><td></td><td>|__|__|.|__|__|</td></tr>
<tr><td rowspan="2">Partial Report (PR)</td><td>PR Score</td><td>|__|__|</td><td></td><td>|__|__|.|__|__|</td></tr>
<tr><td>PR Span</td><td>|__|.|__|__|</td><td></td><td>|__|__|.|__|__|</td></tr>
<tr><td colspan="6">Total time (min)</td><td>51</td></tr>
</table>

<table>
<tr><th>Internal Code</th><th>Patient Number</th><th rowspan="2">Group 1
Training PHONO1/VA2/IM3</th></tr>
<tr><td>19-HPNCL-02</td><td>|__|__| / |__|__| / |__|__|__|__|</td></tr>
</table>

<table>
<tr><th rowspan="38">PHASE 2 TEST 5 V53</th><th colspan="2">Tests</th><th>Data</th><th>Raw scores</th><th>Rate + or -</th><th>Z Scores (SD or percentiles)</th><th>Time (min)</th></tr>
<tr><td rowspan="6">Global/local analysis "SIGL© Drawings"</td><td rowspan="3">Response time (RT)</td><td>Local RT (ms)</td><td>|__|__|__|.|__|__|</td><td></td><td>|__|__|.|__|__|</td><td rowspan="6">20</td></tr>
<tr><td>Global RT (ms)</td><td>|__|__|__|.|__|__|</td><td></td><td>|__|__|.|__|__|</td></tr>
<tr><td>Interference asymmetry RT (ms)</td><td>|__|__|__|.|__|__|</td><td></td><td>|__|__|.|__|__|</td></tr>
<tr><td rowspan="3">Error Rate (ER)</td><td>Local ER (%)</td><td>|__|__|__|.|__|__|</td><td></td><td>|__|__|.|__|__|</td></tr>
<tr><td>Global ER (%)</td><td>|__|__|__|.|__|__|</td><td></td><td>|__|__|.|__|__|</td></tr>
<tr><td>Interference asymmetry ER (%)</td><td>|__|__|__|.|__|__|</td><td></td><td>|__|__|.|__|__|</td></tr>
<tr><td rowspan="6">Word reading "EVALEC©"</td><td rowspan="2">Regular words (RW)</td><td>Accuracy (% errors)</td><td>|__|__|__|.|__|__|</td><td></td><td>|__|__|.|__|__|</td><td rowspan="6">8</td></tr>
<tr><td>Time in ms</td><td>|__|__|__|__|</td><td></td><td>|__|__|.|__|__|</td></tr>
<tr><td rowspan="2">Irregular words (IW)</td><td>Accuracy (% errors)</td><td>|__|__|__|.|__|__|</td><td></td><td>|__|__|.|__|__|</td></tr>
<tr><td>Time in ms</td><td>|__|__|__|__|</td><td></td><td>|__|__|.|__|__|</td></tr>
<tr><td rowspan="2">Non-words (NW)</td><td>Accuracy (% errors)</td><td>|__|__|__|.|__|__|</td><td></td><td>|__|__|.|__|__|</td></tr>
<tr><td>Time in ms</td><td>|__|__|__|__|</td><td></td><td>|__|__|.|__|__|</td></tr>
<tr><td rowspan="2">Surveys-Likert scale</td><td>Child pre-test</td><td>Total points</td><td>|__|__|</td><td></td><td>X</td><td rowspan="2">5</td></tr>
<tr><td>Parents pre-test</td><td>Total points</td><td>|__|__|</td><td></td><td>X</td></tr>
<tr><td rowspan="4">Digit span</td><td rowspan="4">"EVALEO©"</td><td>Score forward span</td><td>|__|</td><td></td><td>|__|</td><td rowspan="4">5</td></tr>
<tr><td>Forward span</td><td>|__|</td><td></td><td>|__|</td></tr>
<tr><td>Score backward span</td><td>|__|</td><td></td><td>|__|</td></tr>
<tr><td>Backward span</td><td>|__|</td><td></td><td>|__|</td></tr>
<tr><td rowspan="2">Visuo-spatial span</td><td rowspan="2">"Corsi©"</td><td>Forward span</td><td>|__|</td><td></td><td>|__|__|.|__|__|</td><td rowspan="2">8</td></tr>
<tr><td>Backward span</td><td>|__|</td><td></td><td>|__|__|.|__|__|</td></tr>
<tr><td rowspan="2">Written comprehension</td><td rowspan="2">"Orlec©, L3"</td><td>Number of correct items</td><td>|__|__|</td><td></td><td>|__|__|.|__|__|</td><td rowspan="2">5</td></tr>
<tr><td>Percentage of correct items</td><td>|__|__|__|.|__|__|</td><td></td><td>|__|__|.|__|__|</td></tr>
<tr><td rowspan="3">Reading meaningless text</td><td rowspan="3">"DeltaText© 1"</td><td>Number of words read</td><td>|__|__|__|</td><td></td><td>X</td><td rowspan="3">3</td></tr>
<tr><td>Number of errors</td><td>|__|__|__|</td><td></td><td>X</td></tr>
<tr><td>Time in seconds</td><td>|__|__|__|</td><td></td><td>X</td></tr>
<tr><td rowspan="3">Words read in 2 min</td><td rowspan="3">"Eval 2M©"</td><td>Number of words read</td><td>|__|__|__|</td><td></td><td>|__|</td><td rowspan="3">2</td></tr>
<tr><td>Number of correct words</td><td>|__|__|__|</td><td></td><td>|__|</td></tr>
<tr><td>% correct/read</td><td>|__|__|__|.|__|__|</td><td></td><td>|__|</td></tr>
<tr><td colspan="6">Total time (min)</td><td>53</td></tr>
</table>

PHASE 3 – NO INTENSIVE TRAINING –TEST 6 – V 54 to 62

Training is interrupted for 2 months but weekly STS continue. A final evaluation is then carried out to check that the gains obtained during training remain stable.

SPEECH THERAPY SESSIONS (V 54 TO V 62)

	DATES (DD/MM/YYYY)	Reading	Spelling
V54	|__|__| / |__|__|/ |__|__|__|__|	❑	❑
V55	|__|__| / |__|__|/ |__|__|__|__|	❑	❑
V56	|__|__| / |__|__|/ |__|__|__|__|	❑	❑
V57	|__|__| / |__|__|/ |__|__|__|__|	❑	❑
In58	|__|__| / |__|__|/ |__|__|__|__|	❑	❑
At59	|__|__| / |__|__|/ |__|__|__|__|	❑	❑
V60	|__|__| / |__|__|/ |__|__|__|__|	❑	❑
V61	|__|__| / |__|__|/ |__|__|__|__|	❑	❑

FOLLOW-UP ASSESSMENT – TEST 6 – V 62

For the administration of the tests, refer to the "testing manuals". Tests must be administered in a random order to be different for each participant.

Date: |__|__| / |__|__| / |__|__|__|__| ***(DD/MM/YYYY)***

- Alouette©
- Delta text© 2
- Eval2M©
- Pingouin©
- Surveys (Likert scale)

Internal Code	Patient Number	Group 1<br>Training PHONO1/VA2/IM3
19-HPNCL-02	I__I__I / I__I__I / I__I__I__I__I	

Phase 3 Test 6 V 62	Tests		Data	Raw scores	Rate + or -	Z Scores	Time min
	Reading meaningless text	"Alouette©"	Number of errors	I__I__I		I__I__I.I__I__I	3
			Number of words read	I__I__I__I		I__I__I.I__I__I	
			Number of correct words	I__I__I__I		I__I__I.I__I__I	
			Time in seconds	I__I__I__I		I__I__I.I__I__I	
			Accuracy index	I__I__I__I		I__I__I.I__I__I	
			Speed index	I__I__I__I		I__I__I.I__I__I	
			Reading age	|__|__| years |__|__| months			
	Reading meaningless text	"DeltaText© 2"	Number of words read	I__I__I__I			3
			Number of errors	I__I__I__I			
			Time in seconds	I__I__I__I			
	Words read in 2 min	"Eval 2M©"	Number of words read	I__I__I__I		|__|	2
			Number of correct words	I__I__I__I		|__|	
			% correct/read	|__|__|__|__|.|__|__|		|__|	
	Reading meaningful text "EVALEO©"	"Pingouin"	Number of words read	I__I__I__I		I__I	2
			Number of correct words	I__I__I__I		I__I	
			% correct/read	I__I__I__I__I.I__I		I__I	
			Degradation Index	I__I__I__I		I__I	
	Surveys-Likert scale	Child pre-test	Total points	I__I__I			5
		Parents pre-test	Total points	I__I__I			
	Total time (min)						16

UNWANTED / SERIOUS / ADVERSE EVENTS since the last visit *(if applicable)*

In this study, no adverse effects are expected since these are non-invasive tests and treatments. However, any occurrence of adverse events will be notified in the case report form and treated in accordance with current regulations.

TREATMENTS

- **Modified specific/experimental** treatment(s) (started, stopped, frequency) since the last check-up
  ↬*If YES, please fill or update* the "Specific treatment sheet/experimental"

- **Modified concomitant** treatments (started, stopped, or frequency) since the last check-up
  ↬*If YES, fill or update the* "Concomitant treatments sheet" (CRF test 1)

Note: The child's academic remediation (related to his pathology) is a concomitant treatment.

Internal Code	Patient Number	Group 1<br>Training PHONO1/VA2/IM3
19-HPNCL-02	I__I__I / I__I__I / I__I__I__I__I	

END-OF-STUDY SHEET

End-of-study date (DD/MM/YYYY): |__|__|/|__|__|/|__|__|__|__|

(last information collected as part of the study: no study-related reviews, treatment administration or data collection will be conducted after that date)

Study followed to completion: ❑ **Yes** ❑ **No**

If "No":

Reason for premature exit:

❑ Refusal to continue/Withdrawal of patient consent

❑ Missing participant

❑ Daily training interrupted for more than 15 days without good cause, subject to the judgment of the investigator

❑ Irregular daily training, subject to the judgment of the investigator

❑ Other (to be specified below)

Accuracy of the reason for premature exit:

………………………………………………… ………………………………………………..

…………………………… ……………………………………………………………………..

Last contact date (DD/MM/YYYY)

Deviation from protocol: ❑ **Yes** ❑ **No**

↳If yes, specify:

....

....

General comments:

………………………………………………………………………………………………………

………………………………………………………………………………………………………

………………………………………………………………………………………………………

Investigator's signature

Internal Code	Centre Nb.	Patient Nb.	SPECIFIC TREATMENT SHEET / EXPERIMENTAL
XX-XX-XX	|__|__|	|__|__|	

	**Treatments**				**Start date** (DD/MM/YYYY)	**End Date** (DD/MM/YYYY) *Note "Ongoing" if the treatment has not been completed at the end of the study*	**Reasons for change or termination**
	**Phases 1, 2 or 3**	**Training 1, 2 or 3**	**PHONO 1 or 2 VA 1 or 2 IM 1 or 2**	**Visits**			
1	… ………	… …………	……….	……….	|__|__| / |__|__| / |__|__|__|__|	|__|__| / |__|__| / |__|__|__|__| ❑in progress	❑ Computer bug<br>❑ Sick leave<br>❑ Vacation<br>❑ Other, specify:... ....
2	… ………	… …………	……….	……….	|__|__| / |__|__| / |__|__|__|__|	|__|__| / |__|__| / |__|__|__|__| ❑in progress	❑ Computer bug<br>❑ Sick leave<br>❑ Vacation<br>❑ Other, specify:... ....
3	… ………	… …………	……….	……….	|__|__| / |__|__| / |__|__|__|__|	|__|__| / |__|__| / |__|__|__|__| ❑in progress	❑ Computer bug<br>❑ Sick leave<br>❑ Vacation<br>❑ Other, specify:... ....
4	… ………	… …………	……….	……….	|__|__| / |__|__| / |__|__|__|__|	|__|__| / |__|__| / |__|__|__|__| ❑in progress	❑ Computer bug<br>❑ Sick leave<br>❑ Vacation<br>❑ Other, specify:... ....
5	… ………	… …………	……….	……….	|__|__| / |__|__| / |__|__|__|__|	|__|__| / |__|__| / |__|__|__|__| ❑in progress	❑ Computer bug<br>❑ Sick leave<br>❑ Vacation<br>❑ Other, specify:... ....
