# Supplementary material for: Multimodal intervention in 8- to 13-year-old French dyslexic readers: Study protocol for a randomized multicenter controlled crossover trial
Source: BMC Pediatr. 2022 Dec 28;22:741. doi: 10.1186/s12887-022-03701-8 (PMC9795620; doi:10.1186/s12887-022-03701-8)
Supplement: Supplementary file 8 — Additional file 8. Likert scale-child-parent (Assessment of reading disability impact). [file 12887_2022_3701_MOESM8_ESM.pdf]

Assessment of reading disability impact

PRE-TEST LIKERT SCALE - CHILD						
	**1** Not at all	**2** A little	**3** Average	**4** A lot	**5** Very much	**Total**
I like to read						
Reading is easy						
I read quickly						
I've already read several books from beginning to end						
Outside of school and homework, I read other books or magazines						
Outside of school and homework, I read comics						
I understand what I'm reading						
I like to get a book as a present						
I have good grades in French (or English)						
I have good grades in History/Geography						
I have good grades in Mathematics						
I think I can do well in school						
I like to go to school						
Total score						

PRE-TEST LIKERT SCALE - PARENTS						
	**1** Not at all	**2** A bit	**3** Normal	**4** A lot	**5** So much	**Total**
Your child likes to read.						
Reading is an easy activity for him/her.						
He/she reads quickly.						
He/she has already read several books entirely.						
Outside of school, he/she reads other books or magazines.						
Outside of school, he/she reads comics.						
Does your child understand what is he/she reading ?						
Does he/she appreciate a book as a gift ?						
Does he/she have good grades in French (or English)						
Does he/she have good grades in History/Geography ?						
Does he/she have good grades in Mathematics ?						
Does he/she think he or she can do well in school ?						
Does he/she enjoy going to school ?						
Total score						

POST-TEST LIKERT SCALE - CHILD						
	**1** Not at all	**2** A little	**3** Average	**4** A lot	**5** Very much	**Total**
I like to read						
Reading is easy						
I read quickly						
I've already read several books from beginning to end						
Outside of school and homework, I read other books or magazines						
Outside of school and homework, I read comics						
I understand what I'm reading						
I like to get a book as a present						
I have good grades in French (or English)						
I have good grades in History/Geography						
I have good grades in Mathematics						
I think I can do well in school						
I like to go to school						
Total score						

POST-TEST LIKERT SCALE - PARENTS						
	**1** Not at all	**2** A bit	**3** Normal	**4** A lot	**5** So much	**Total**
Your child likes to read.						
Reading is an easy activity for him/her.						
He/she reads quickly.						
He/she has already read several books entirely.						
Outside of school, he/she reads other books or magazines.						
Outside of school, he/she reads comics.						
Does your child understand what is he/she reading ?						
Does he/she appreciate a book as a gift ?						
Does he/she have good grades in French (or English)						
Does he/she have good grades in History/Geography ?						
Does he/she have good grades in Mathematics ?						
Does he/she think he or she can do well in school ?						
Does he/she enjoy going to school ?						
Total score						
